# *In silico* predictions of drug-induced changes in human cardiac contractility align with experimental recordings

**DOI:** 10.3389/fphar.2025.1500668

**Published:** 2025-03-17

**Authors:** Cristian Trovato, Stefano Longobardi, Elisa Passini, Kylie A. Beattie, Maxx Holmes, Khuram W. Chaudhary, Eric I. Rossman, Blanca Rodriguez

**Affiliations:** ^1^ Department of Computer Science, University of Oxford, Oxford, United Kingdom; ^2^ Systems Medicine, Clinical Pharmacology and Safety Science, R&D, AstraZeneca, Cambridge, United Kingdom; ^3^ Non-Clinical Safety, Pre-Clinical Sciences, GlaxoSmithKline, Stevenage, United Kingdom; ^4^ Non-Clinical Safety, Pre-Clinical Sciences, GlaxoSmithKline, Upper Providence, Collegeville, PA, United States

**Keywords:** cardiac contractility, drug safety, systems toxicology, cardiac modelling, human cardiomyocytes, human modelling

## Abstract

Drug-induced changes in cardiac contractility (inotropy) can lead to cardiotoxicity, a major cause of discontinuation in drug development. Preclinical approaches to assess cardiac inotropy are imperfect, with *in vitro* assays limited to stem cell-derived or adult human primary cardiomyocytes. Human mechanistic *in silico* modelling and simulations are already successfully applied for proarrhythmia prediction, contributing to cardiac safety assessment strategies in early drug development. In this study, we investigated their ability to predict drug-induced effects on cardiac inotropy. We considered a validation set of 28 neutral/negative inotropic and 13 positive inotropic reference compounds and simulated their effects on cell contractility via ion channel inhibition and perturbation of nine biomechanical modelling parameters, respectively. For each compound, a wide range of drug concentrations was simulated in an experimentally calibrated control population of 323 human ventricular *in silico* cells. Simulated biomarkers indicating drug-induced inotropic effects were compared with *in vitro* preclinical data from the literature. Computer simulations predicted drug-induced inotropic changes observed *in vitro* for 25 neutral/negative inotropes and 10 positive inotropes. Predictions of negative inotropic changes were quantitatively in agreement for 86% of tested drugs. Active tension peak was identified as the biomarker with highest predictive potential. This study describes the validation and application of an *in silico* cardiac electromechanical model for drug safety evaluation, combining ion channel inhibition data and information on potential inotropic mechanisms to predict inotropic changes. Furthermore, a route for its integration as part of a preclinical drug safety assessment strategy is outlined.

## 1 Introduction

Drug-induced changes in cardiac contractility (inotropy) can lead to cardiotoxicity, a major cause of discontinuation of drug development projects (Klein and Redfern, 2015; Mamoshina et al., 2021). Accurate assessment of drug-induced effects on cardiac inotropy during pre-clinical stages of drug development remains challenging, without a consensus on gold standard biomarkers for comparison of *in vitro* results to *in vivo* or clinical biomarkers for model validation. In addition, non-clinical *in vitro* strategies to assess changes in contractility for both small and large molecules are limited to low/medium throughput assays, which do not always translate to clinical outcomes. The most common *in vitro* human models comprise stem cell-derived cardiomyocytes (Pointon et al., 2015) and adult human primary cardiomyocytes (Abi-Gerges et al., 2020; Nguyen et al., 2017). Despite both models offering valuable insights into human heart function and pathology, they present important limitations: stem cell-derived cardiomyocytes have an immature phenotype, which impacts the Ca^2+^ dynamics, leading to less robust predictions. Adult human primary cardiomyocytes have short lifespans in culture, lack proliferative capacity, are not readily available, pose ethical and legal considerations, and are expensive and difficult to handle. Therefore, alternative approaches should be considered to address some of the gaps in current cardiac contractility assessment strategies, particularly in the context of developing a high-throughput framework to inform dosing strategies for *in vivo*/clinical studies.

Cardiac contraction is initiated by an increase in the intracellular Ca^2+^ concentration ([Ca^2+^]_i_). Ca^2+^ binds to troponin C on the thin filament, which causes tropomyosin to move out of the actin groove, exposing actin-binding sites. The thick filament, composed of many myosin molecules, has a central core of aligned myosin tails with protruding myosin heads; these myosin heads bind to the exposed actin-binding sites. Contraction then follows, as described by the *sliding filament theory* (Huxley, 1953). The attached myosin heads rotate in the power stoke, pulling the thick filaments past the thin filaments and causing the sarcomere to contract. The myosin heads then unbind and can reattach to actin to further contract the sarcomere (Lewalle et al., 2022).

The sarcomere is situated within the selectively permeable cell membrane, which aids in maintaining intracellular ionic homoeostasis. Within this highly regulated space, disruption in Ca^2+^ homoeostasis will impact normal contraction and relaxation. Ca^2+^ diffuses from the dyadic space into the cytosol, which triggers further release of Ca^2+^ from an intracellular Ca^2+^ store called the sarcoplasmic reticulum (SR), and it then binds to the sarcomeric proteins, activating contraction. As Ca^2+^ is removed from the cytosol, [Ca^2+^]_i_ decreases, causing Ca^2+^ to dissociate from the sarcomeric proteins and leading to sarcomere relaxation. Ca^2+^ removal is achieved either through Ca^2+^ extrusion from the cell or via Ca^2+^ uptake into the SR (Hall and Hall, 2021; Levick, 2009).

Block of the L-type Ca^2+^ channel and subsequent binding to the ryanodine receptors (Ca^2+^-release channels located on the SR) will prevent Ca^2+^ diffusion from the dyadic space into the cytosol from the SR. At the same time, blocking the sodium–calcium exchanger (NCX), the plasma membrane Ca^2+^ ATPase (PMCA), or the SR Ca^2+^ ATPase (SERCA) will prevent Ca^2+^ extrusion from the cytosolic space. Sarcomere modulators are emerging as an important class of compounds since the dynamics of sarcomeric proteins form the foundation of myocardial contraction and relaxation. Sarcomere modulators can alter myofilament Ca^2+^ sensitivity without altering Ca^2+^ homoeostasis (Longobardi et al., 2022).

In the last decade, *in silico* approaches using human-based, biophysically detailed models and multiscale simulations have proven to be powerful tools for drug safety assessment, particularly for predicting proarrhythmic risk (Lancaster and Sobie, 2016; Passini et al., 2017; Li et al., 2019a; Passini et al., 2019; Llopis-Lorente et al., 2022; Trovato et al., 2022). Human-based electromechanical models have also been recently published for simultaneous proarrhythmic and inotropic risk assessment of drug action on ion channels and cross-bridge dynamics (Margara et al., 2021). The use of *in silico* approaches has been supported by regulators such as the United States Food and Drug Administration (FDA), which led the Comprehensive *in vitro* Proarrhythmia Assay (CiPA) initiative (Sager et al., 2014; Li et al., 2019b), and the European Medicines Agency (Musuamba et al., 2021), which established a task force on medical innovation to facilitate the adoption of innovative products, methods, and technologies in drug development. One of the main achievements of these initiatives was the identification of general principles for model design, development, and validation (Musuamba et al., 2021; Li et al., 2019c), which go beyond proarrhythmia prediction and can be applied to different contexts of use.

**FIGURE 1:**
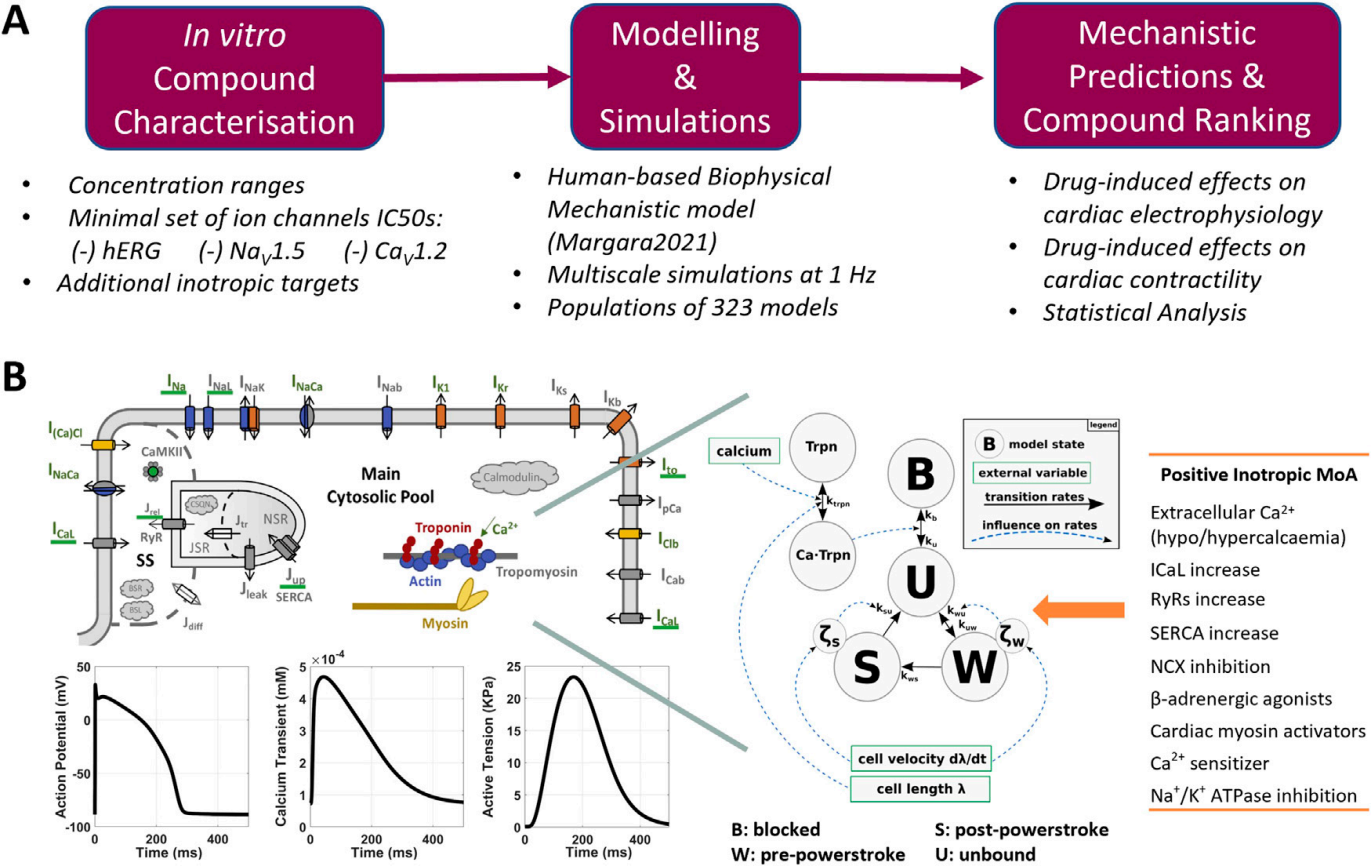
**(A)** Proposed pipeline for the *in silico* assessment of drug-induced changes in human cardiac electrophysiology and contractility. **(B)** Modelling and simulation overview: structure of the Margara2021 model, combining the ToR-ORd model for human cardiac electrophysiology (top left) and the Land model for cardiomyocyte mechanics description (centre), as described by Margara et al. (2021). Exemplificative model outputs (bottom-left) and the list of modes of action (MoA) tested to simulated positive inotropic compounds (right) are also shown.

In this study, we investigated the feasibility of predicting and explaining drug-induced effects on cardiac cellular inotropy, action potential, and calcium dynamics using mechanistic, *in silico* human multiscale modelling and simulations, with *in vitro* ion channel measurements and known inotropic modes of drug action as input (Figure 1A). First, we simulate hypothetical specific Na^+^, K^+^, and Ca^2+^ channel blockers to assess model sensitivity and identify the most informative biomarkers. Then, a set of 41 reference compounds was considered a validation dataset. Among these compounds, 28 inhibit specific cardiac ion channels and exhibit negative or non-inotropic effects. Half-maximal inhibitory concentration (IC_50_) values were used to describe the drug-induced effects on ionic currents and served as a model input. For each compound, a wide range of drug concentrations was simulated in an experimentally calibrated population of 323 human ventricular *in silico* cells, representing a healthy control population. For the remaining 13 compounds, the known modes of action are more heterogeneous, thus requiring an additional explorative step to be applied using the same *in silico* framework. Simulated biomarkers were then compared with *in vitro* preclinical data available in the literature and clinical observations of drug-induced inotropy effects for some compounds. The whole study pipeline is depicted in Figure 1A.

## 2 Materials and methods

### 2.1 *In vitro* measurements of inotropy and set of compounds

Two datasets of *in vitro* measurements of cardiac contractility (Nguyen et al., 2017; Abi-Gerges et al., 2020) were considered to be preclinical *in vitro* evidence of drug-induced inotropic effects. Both datasets comprise optical recordings of sarcomere shortening from isolated human adult primary cardiomyocytes. The list of compounds and concentrations simulated in the present study from each *in vitro* dataset is reported in Table 1. In the first study, Nguyen and others investigated contractility and proarrhythmia biomarkers for CiPA compounds (Li et al., 2019a; Sager et al., 2014), resulting in a collection of negative or neutral inotropic compounds. Drugs were tested in 3–8 samples from 1 or 2 donor hearts. In the second study, Abi-Gerges and others assessed a set of 12 contractility parameters for compounds, leading to positive inotropic effects through a variety of mechanisms of action, including hypo/hypercalcaemia, I_CaL_ increase, ryanodine receptor (RyR) modulation, SERCA modulation, NCX inhibition, β-adrenergic stimulation, adenylyl cyclase activation, phosphodiesterase (PDE) inhibition, cardiac myosin activation, Ca^2+^ sensitization, and Na^+^/K^+^ ATPase inhibition. We will refer to the compound data from the first and second studies as “Dataset 1” and “Dataset 2,” respectively, throughout.

**TABLE 1:** *In vitro* datasets used for comparison against model predictions: number of drugs simulated in this study, concentration ranges tested *in vitro*, and compound names.

	No. of drugs	Concentration tested	Compound
Dataset 1 (Nguyen et al., 2017)	28	Multiple of EFTPC [0.1x; 222x]	Astemizole, bepridil, chlorpromazine, cisapride, clarithromycin, clozapine, sotalol, diltiazem, diphenhydramine, disopyramide, dofetilide, domperidone, droperidol, erythromycin, flecainide, ibutilide, loratadine, mexiletine, mibefradil, moxifloxacin, nifedipine, nitrendipine, ondansetron, procainamide, quinidine, ranolazine, sematilide, tamoxifen, terodiline, vandetanib, and verapamil
Dataset 2 (Abi-Gerges et al., 2020)	13	See Supplementary Table S2 in the original study	CaCl_2_, Bay-K 8644, caffeine, N106, SEA-0400, isoproterenol, epinephrine, dobutamine, omecamtiv mecarbil, EMD-57003, levosimendan, digoxin, and ouabain

EFTPC, effective free therapeutic plasma concentration.

Combining selected compounds (see Section 2.2) from both *in vitro* datasets, we obtained a collection of 41 compounds, of which 23 led to a negative inotropic effect, 13 to a positive inotropic effect, and 5 had no effects on contractility. A complete description of the *in vitro* experiments can be found in the original articles (Nguyen et al., 2017; Abi-Gerges et al., 2020).

### 2.2 *In vitro* IC_50_ values of ion channel inhibition for simulating negative inotropic compounds

Drug-induced changes in cardiac electrophysiology are routinely screened in the early stage of drug development, either as primary (on-target) or secondary (off-target) pharmacological signals. The most important signal is hERG since its inhibition can lead to QT prolongation and Torsades de Pointes arrhythmia; therefore, hERG screening is a regulatory requirement (as detailed in ICH S7B). Other pharmacological signals include *in vitro* recordings of other cardiac ion currents, such as the fast sodium current (I_Na_), the late sodium current (I_NaL_), the L-type calcium current (I_CaL_), the slow potassium rectifier (I_Ks_), the outward potassium current (I_to_), and the potassium inward rectifier (I_K1_). However, not all of these currents might be routinely screened during early drug development, and not all of them may be crucial for safety assessment (Zhou et al., 2020).

In this study, to simulate inotropic changes induced by the compounds in Dataset 1, i.e., non-inotropic or negative inotropic compounds, we used IC_50_ values, Hill coefficients, and free therapeutic plasma concentrations available in the literature (Crumb et al., 2016; Delaunois et al., 2021; Kramer et al., 2013; Passini et al., 2019) and listed in Supplementary Table S1. Most compounds are characterised by IC_50_ values and Hill coefficients for I_Kr_, I_Na_, and I_CaL_, similar to what would happen during the early stages of drug development (Figure 1A). For five compounds, namely, ajmaline, azimilide, sematilide, diphenhydramine, and tamoxifen, no homogenous (i.e., recorded as much as possible in the same experimental settings and from the same source) IC_50_ values were available in the literature; therefore, these compounds were excluded from this study. For the same reason, two negative inotropic compounds from Dataset 2 (ryanodine and thapsigargin) were excluded from this study.

### 2.3 Human modelling and simulation framework

#### 2.3.1 *In silico* electromechanical modelling for human ventricular cardiomyocytes

The Margara2021 model (Margara et al., 2021) was selected as the *in silico* model for simulating electromechanical dynamics in human ventricular cardiomyocytes (Figure 1B). The main outputs of the Margara2021 model are action potential (AP), intracellular calcium transient (CaT), and active tension (AT) time courses over a single beat or multiple beats at a given pacing frequency. Representative traces (1 beat at 1 Hz pacing frequency) are shown in Figure 1B.

The Margara2021 model combines the human ventricular electrophysiological ToR-ORd cellular model (Tomek et al., 2019) with a model of human cardiomyocyte mechanics (Land et al., 2017), both calibrated and validated with experimental data. In addition to the already mentioned I_Na_, I_CaL_, I_Kr_, I_Ks_, I_NaL_, I_K1_, and I_to_, the ToR-ORd model in the Margara2021 model also includes a mathematical description for the sodium–calcium exchange current (I_NCX_), the sodium–potassium ATPase current (I_NaK_), the sarcolemmal calcium pump current (I_pCa_), and sodium (Na^+^) and potassium (K^+^) background currents. The model also incorporates a detailed description of the calcium subsystem and excitation–contraction coupling, including calcium release from the ryanodine receptors, calcium uptake through SERCA pumps, calcium buffers (calmodulin, troponin, anionic SR and sarcolemmal-binding sites, and calsequestrin), and calcium-/calmodulin-dependent protein kinase.

The Land model is based on measurements obtained from human cardiomyocytes at body temperature, and it comprises a three-state cross-bridge model to reproduce troponin C and tropomyosin kinetics, accounting for cross-bridge distortion. The bidirectional electromechanical coupling between ToR-ORd and Land models in the Margara2021 model is based on the free [Ca^2+^]_i_ computed in the ToR-ORd electrophysiology model, which serves as the input for the Land model. In turn, the amount of Ca^2+^ bound to troponin C, computed in the Land model, is fed back into the ToR-ORd electrophysiological model and used to update the free [Ca^2+^]_i_.

#### 2.3.2 Simulating human cardiac electromechanical dynamics in a population of models

Using the Margara2021 model, we developed an experimentally calibrated population of human cardiac ventricular cardiomyocytes, incorporating cell-to-cell variability. The population was designed as in previous studies (Passini et al., 2017; Passini et al., 2019; Trovato et al., 2020; Trovato et al., 2022), following the population of models methodology (Britton et al., 2013; Lancaster and Sobie, 2016; Muszkiewicz et al., 2015). The population was constructed using Virtual Assay software (v.3.2 ^©^ 2018 Oxford University Innovation Ltd. Oxford, UK), a user-friendly software program to perform *in silico* simulations in a population of models (Passini et al., 2021).

An initial population of 1,000 models was constructed by sampling nine conductance values of the main ionic currents mentioned above (I_Na_, I_NaL_, I_CaL_, I_to_, I_Kr_, I_Ks_, I_K1_, I_NCX_, and I_NaK_) and Ca^2+^ uptake and release maximal currents in the range [50–150]% of their baseline values. All these models were paced individually at 1 Hz for 500 beats to allow the models to reach Na^+^, K^+^, and Ca^2+^ diastolic concentration stability (steady state), and the last-beat output traces for each model were used to compute a set of 15 biomarkers. In particular, seven biomarkers characterised the AP curve: AP duration at 40%, 50%, and 90% of repolarisation (APD_40_, APD_50_, and APD_90_); AP triangulation, defined as the difference between APD_90_ and APD_40_ (Tri90-40); maximum upstroke velocity (dV/dt_max_); peak voltage (V_peak_); and resting membrane potential (RMP); four biomarkers characterised the CT curve: duration at 50% and 90% of repolarisation (CTD_50_ and CTD_90_) and minimum/diastolic and maximum calcium concentrations (CaiD and CT_peak_); and four biomarkers characterised the AT curve: AT peak (AT_peak_), AT time to peak (AT_ttp_), and AT relaxation times at 50% and 90% (ATrt_50_ and ATrt_90_). In addition to the abovementioned biomarkers, the electromechanical window (EMw, Passini et al., 2019) was computed as the difference between CTD_90_ and APD_90_, and qNet was computed as the total net charge (i.e., balancing inward and outward currents) flowing through I_NaL_, I_CaL_, I_to_, I_Kr_, I_Ks_, and I_K1_ over an entire beat (Chang et al., 2017).

The population was then filtered based on biomarker values (Supplementary Table S2) from healthy human left ventricular myocytes (Margara et al., 2021; Passini et al., 2019), and a total of 322 models (out of the initial 1,000 models) whose biomarkers were within experimental ranges were retained. These 322 models constituted the experimentally calibrated population and were then used for the simulations, along with the baseline model, resulting in a total of 323 electrophysiological profiles.

Running a full population simulation involves concurrently running the 323 models using their calibrated ion channel parametrisations. Each model is paced for 1,000 beats at 1 Hz, and the last-beat curves (AP, CaT, and AT) are regarded as the model outputs. The simulated AP curve is then checked for the occurrence of depolarisation and repolarisation abnormalities, as defined by Passini et al. (2017): i) repolarisation abnormalities were defined as the presence of a positive derivative of the membrane potential following V_peak_ (early after depolarisation) or when the membrane potential did not reach the resting condition by the end of the beat (repolarisation failure); ii) depolarisation abnormalities occurred when the upstroke phase was compromised, i.e., when V_peak_ was lower than 0 mV or when the time needed to reach 0 mV was longer than 100 ms. Finally, the biomarkers described above are calculated from the output curves for all models where there was no prediction of abnormalities.

Drug effects can be incorporated by further manipulating the baseline parametrisation of all the population models for the ion channels and/or other mechanisms before running a simulation. In the next two sections, we will describe in detail how we performed simulations for the compounds from experimental Dataset 1 and Dataset 2.

#### 2.3.3 Incorporating drug effects via dose-dependent ion channel inhibition

A simple pore-block model (Brennan et al., 2009) was used to simulate ion channel inhibition under a compound effect. This model provides the fraction of residual current $I_{res}$ as a function of compound-specific binding affinity parameters (the ${IC}_{50}$ value and Hill coefficient) at any given compound concentration C :

$$I_{res}=\frac{1}{1+{\left(\frac{C}{{IC}_{50}}\right)}^{Hill}}$$

For each compound in Dataset 1, we tested a wide range of concentrations, taken as multiples of the compound’s EFTPC, ranging from 0.1× to 100×. This range extended well beyond the concentrations estimated/clinically measured for humans, allowing for a broader exploration of any drug-induced effects on electrophysiology and contractility. One of the main advantages of using *in silico* modelling is that we can explore a large number of concentrations, which would be unfeasible to test experimentally, although caveated with further modelling assumptions, such as neglecting the interplay of solubility and metabolism. Therefore, for those compounds that did not reach a 50% reduction in peak tension, we additionally extended the concentration range up to 100,000× the EFTPC to check for saturating behaviours, for model verification purposes.

**FIGURE 2:**
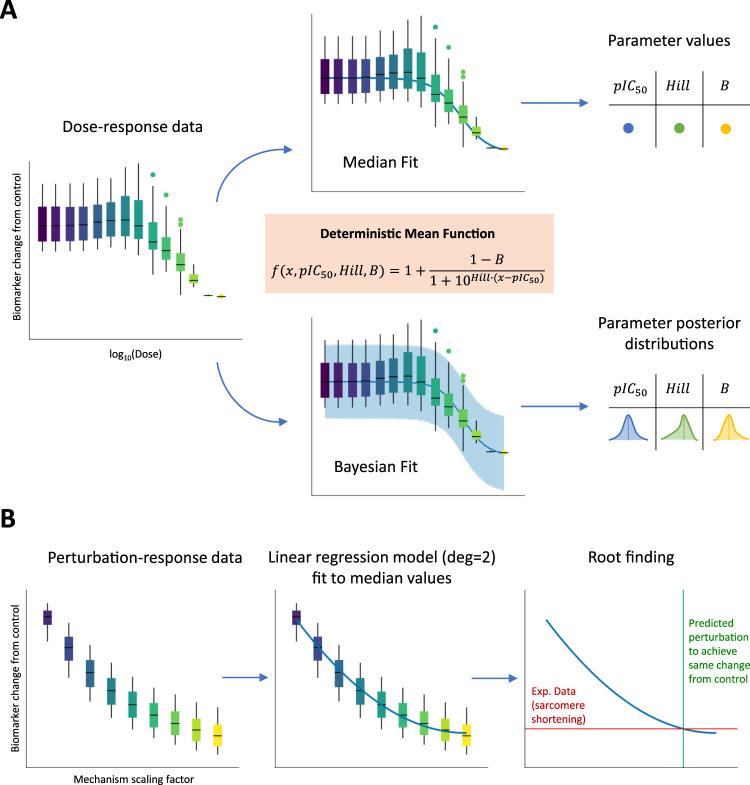
Fitting dose–response and perturbation-response curves to biomarker values simulated using the full population of models. **(A)** The three main parameters (including pIC_50_ and Hill values) of a sigmoid function (orange box) were fitted using two approaches: (1-top panel) non-linear least-squares method to minimize the distance of the curve from median population biomarker values at the tested concentrations and (2-bottom panel) a Bayesian method to incorporate population biomarker values’ variability. In the first case, pointwise estimates are derived for the three sigmoid parameters, while in the second case, full posterior distributions are derived instead. **(B)** A linear regression model with degree equal to 2 (parabola) was fitted to median population biomarker values at the different tested mechanism perturbations. An experimental sarcomere shortening value observed at the EC_50_ value for drugs represented by the mechanism is then projected on the parabola to find the intersection point corresponding to the mechanism scaling factor needed to reproduce *in silico* the experimental observation.

For each drug, dose–response curves were derived for the peak tension biomarker. We used two methods to evaluate the impact of different metrics on inotropy assessment: (1) a classic non-linear least squares approach to fit a Hill curve through median biomarker values from the full, simulated population and (2) a Bayesian approach to fit the same type of curve by incorporating biomarker variability across the full population of models. Figure 2A illustrates the fitting process. In the first case using biomarker medians, single ${IC}_{50}$ and Hill values are obtained from the sigmoid fit. In the second case, using all biomarker values, full posterior distributions for the same parameters are derived instead. We employed the same Bayesian framework as described by Labelle et al. (2019). In brief, we used a normal likelihood with a sigmoid deterministic mean function and an isotropic Gaussian noise. Weakly informative normal priors were used for all parameters, providing gentle constraints that allow the data to dominate the inference. For the likelihood noise, a half-normal prior was used to ensure non-negativity while maintaining flexibility. The isotropic Gaussian noise assumption was specifically chosen because the same 323 computer models were run at each concentration, providing a consistent framework for comparing responses. This setup implies that the variability observed at each concentration arises from independent and identically distributed noise across the models, rather than from heteroscedasticity or concentration-dependent correlation structures. By assuming constant variance and no correlation, the model captures the intrinsic randomness in responses while avoiding the need to introduce additional parameters to describe noise patterns that are not evident in the data. From a performance and robustness perspective, this assumption ensures that the Bayesian framework can focus on characterising the true variability across the model population without being confounded by unnecessary complexity. It also simplifies the computational process as the residual variance is treated uniformly across concentrations. Finally, a Markov Chain Monte Carlo approach (No-U-Turn sampler) was used to derive posterior distributions. It is worth noting that the ${IC}_{50}$ parameter was not fitted directly; instead, its negative logarithm in base 10, the so-called ${pIC}_{50}$ , was fitted and then converted back into the original units. A parameter called *B* was also included (and fitted) in the model to account for the saturation level of the negative inotropic effect at high concentrations. Unlike traditional Hill-type models, where the response asymptotes are often fixed to 0 by default, *B* provides the flexibility to fit the observed data without imposing a hard constraint on the lower asymptote. This is particularly relevant for negative inotropes as experimental data often indicate a non-zero saturation level that depends on the pharmacological characteristics of the compound. By fitting *B*, the model ensures that the predicted response aligns more accurately with the experimental data, even at very high concentrations, thereby improving the biological and mechanistic interpretability of the curve. In practical terms, after fitting the curve for the set of compounds under investigation, we found that (see Supplementary Figure S6; Supplementary Table S3) all compounds identified as clearly negative inotropes exhibited *B* values very close to 0 (in the Bayesian case, the posterior distribution of *B* was observed to be very narrow around 0 for the same compounds). This suggests that *B* effectively captures the experimentally observed saturation behaviour while allowing flexibility in cases where deviations might exist. The convergence of the posterior distributions was assessed by visually inspecting the trace plots of the four chains (see Supplementary Figure S6) to ensure they were well-mixed and overlapping, which is a standard preliminary check in Bayesian analysis. All the IC_50_ values fitted using both the approaches and enabling model simulations to match neutral/negative inotropic compound effects are reported in Table 2.

**TABLE 2:** Contractility IC_50_ values observed *in vitro* for sarcomere shortening and simulated for the active tension peak. The latter was computed both from median concentration-response values and using a Bayesian approach. The IC_50_ and EFTPC_max_ ratio is reported between brackets.

Compound	*In vitro* IC_50_ in µM (IC_50_/EFTPC_max_ ratio)	*In silico* IC_50_ in µM (IC_50_/EFTPC_max_ ratio)		
		Median	Bayesian	
			Mean	STD
Bepridil	0.7 (22)	0.77 (22)	0.84 (24)	0.06
Chlorpromazine	1.02 (28)	2.5 (69)	2.9 (80)	0.13
Clarithromycin	16 (13)	3,336 (2,780)	2,795 (2,330)	251
Clozapine	1.5 (21)	1.5 (21)	1.6 (23)	0.07
Diltiazem	1 (8)	0.28 (2.3)	0.3 (2.46)	0.0077
Disopyramide	9.3 (13)	407 (549)	468 (631)	27
Domperidone	0.2 (10)	5 (250)	11 (542)	2.4
Droperidol	0.18 (11)	1.5 (95)	1.8 (114)	0.12
Flecainide	1.1 (2)	7.9 (14)	10 (19)	1.7
Ibutilide	2 (20)	16 (155)	27.8 (278)	10
Loratadine	0.017 (35)	5.6 (11,000)	5.7 (11,000)	0.34
Mexiletine	0.9 (0.4)	8.7 (3.5)	9.9 (4)	1.1
Mibefradil	0.18 (13)	0.28 (20)	0.30 (22)	0.01
Nifedipine	0.04 (5)	0.0038 (0.48)	0.0041 (0.51)	0.0001
Nitrendipine	0.06 (18)	0.0054 (1.6)	0.006 (1.8)	0.0003
Ondansetron	14 (34)	7.4 (18)	8.02 (19)	0.24
Procainamide	2,215 (38)	138 (2.4)	157 (2.7)	12
Quinidine	3.6 (1)	2.06 (0.64)	2.3 (0.72)	0.15
Ranolazine	17 (9)	61 (31)	62 (32)	12
Terodiline	0.7 (5)	2.4 (16)	2.5 (17)	0.11
Vandetanib	2.7 (9)	5.2 (17)	6.3 (21)	0.35
Verapamil	0.04 (2)	0.08 (4)	0.08 (4)	0.002

IC_50_, half-maximal inhibitory concentration; EFTPC_max_, maximum effective free therapeutic plasma concentration; STD, standard deviation; µM, micromolar.

#### 2.3.4 Incorporating drug effects via dose-independent mechanism perturbation

To investigate how perturbation of additional mechanisms may affect contractility, a one-at-a-time sensitivity analysis was performed on the Margara2021 model. We selected nine parameters that could be altered in the model to mimic functional effects induced by the positive inotropic compounds (Dataset 2) tested by Abi-Gerges and collaborators on human cardiomyocytes. The selected mechanisms were (1) extracellular Ca^2+^ concentration increase; (2) I_CaL_ activation; (3) RyR activation; (4) SERCA pump activation; (5) Na^+^/Ca^2+^ exchanger inactivation; (6) β-adrenergic stimulation; (7) Ca^2+^ sensitivity decrease; (8) Na^+^/K^+^ pump inactivation; and (9) cardiac myosin activation. Phosphodiesterase inhibitors (IBMX and milrinone) and adenylyl cyclase activators (forskolin and NKH-477) could not be represented using available mechanisms in the Margara2021 model and, therefore, were not included in this simulation study.

Selected model parameters were varied to represent these modulations and are listed in Table 3 *.* The scaling factors for these parameters were defined in a dose-independent fashion to qualitatively and quantitatively reproduce the positive inotropic effects observed *in vitro*. Baseline parameter values were either increased (from 1x to 3x–5x) or decreased (from 1x to 0.1x), according to whether the selected direction of change would correspond to an increase in active tension, with ranges chosen arbitrarily. For each simulated mechanism representing one or multiple drugs, perturbation-response curves were derived for the peak tension biomarker. Given that the scaling factors for each mechanism were varied in a dose-independent fashion, the perturbation-response curves were not ensured to follow sigmoid trends. For this reason, to still capture the non-linear nature of these curves, we used a parabolic fit through the median biomarker values from the full, simulated population. Figure 2B illustrates the fitting process. After fitting, we projected the sarcomere shortening value observed experimentally at the EC_50_ value for the compounds represented in the model by the mechanisms under analysis and found the intersection point (if any existed) with the parabola. This intersection point corresponded to the exact perturbation that had to be performed to the model parameters to achieve the same variation in contractility, as observed experimentally. All the scaling factors calculated using this procedure and enabling model simulations to match positive inotropic compound effects are reported in Table 3.

**TABLE 3:** Comparison between *in vitro* positive inotropic effects and *in silico* mechanism perturbation responses for several modes of drug action: mechanisms of positive inotropy considered in this study, compounds tested *in vitro* by Abi-Gerges et al. (2020), model parameters changed *in silico*, scaling factor ranges, maximum sarcomere shortening observed *in vitro*, maximum active tension variation reached *in silico*, sarcomere shortening observed at the EC_50_ value, and SF to simulate similar drug-induced inotropic effects observed at the EC_50_ value *in vitro.*

Main known MoA	Compound tested *in vitro*	Parameter changed *in silico*	Parameter SF range *in silico*	Max sarcomere shortening^a^ *in vitro*	Max active tension^a^ *in silico*	Sarcomere shortening at EC_50_ in vitro	SF to reproduce *in silico* observed contractility changes at the EC_50_ value *in vitro*
Extracellular Ca^2+^ modulator	CaCl_2_	Cao	[1, 3]	220%	362%	120%	1.24
Ca^2+^ sensitizer	Levosimendan	Ca50	[0.1, 1]	131%	414%	115%	0.85
ICaL activator	Bay-K 8644	GCaL	[1, 3]	180%	375%	138%	1.17
NCX inhibitor	SEA-0400	GNCX	[0.1, 1]	168%	452%	136%	0.79
Na^+^/K^+^ ATPase inhibitor	Digoxin	GNaK	[0.1, 1]	250%	121%	177%	—
	Ouabain			237%		173%	—
Myosin activator	EMD-57003	Kuw	[1, 5]	368%	242%	237%	4.53
	Omecamtiv Mecarbil			263%		181%	2.63
RyR activator	Caffeine	Jrel	[1, 3]	261%	118%	188%	—
SERCA activator	N-106	Jup	[1, 3]	148%	133%	124%	1.62
β-adrenergic agonist	Dobutamine	(GKs, GCaL)	[1, 3] x [1, 3]	226%	450%	171%	1.22
	Isoproterenol			434%		258%	1.56
	Epinephrine			280%		193%	1.30

^a^ mean percentile variation from control. The dash symbol indicates the case when a scaling factor that reproduces experimental observation could not be identified using the proposed modelling and simulation approach.

MoA, mode of action; SF, scaling factor; EC_50_, half-maximal effective concentration; Kuw, cross-binding rate; G_X_, maximal conductance/permeability of channel X; ca50, calcium sensitivity. ‘Cao, extracellular calcium concentration’, Jrel, calcium release from RyR receptors; Jup, calcium uptake by SERCA. Also, Gx, put ‘x’ as normal text, not subscript given that in the table the channels are not subscripted. Also, insert the word ‘ion’ in the phrase: maximal conductance/permeability of ion channel X.

### 2.4 Verification, validation, and uncertainty quantification

We define our verification, validation, and uncertainty quantification strategy based on the principles outlined for the CiPA *in silico* strategy by Li et al. (2019c), the ‘model validation flow’ described by Musuamba et al. (2021) (Figure 1), and the ASME V&V40 standard as applied to *in silico* trials (Viceconti et al., 2021) as follows.

The context of use is the assessment of drug-induced changes in contractility through ion channel modulation and the determination of the mechanisms of inotropy. This framework has previously been validated for proarrhythmic risk assessment (Passini et al., 2017); thus, the verification of software was conducted by ensuring agreement (Supplementary Figure S2) between simulation outputs using Virtual Assay software, the MATLAB code provided by Margara et al. (2021), and CellContraction.jl, the Julia package used in this study and available open source (see Data Availability Statement). The comparator dataset and algorithms utilised are described in Sections 2.1, 2.3, respectively. The criteria for assessing the predictivity of the simulations are defined in Section 2.3. Simulated outputs are validated through comparison with experimental recordings of drug-induced inotropic behaviour in adult human primary cardiomyocytes. Uncertainty quantification of the cellular model was addressed through a population of models approach and the comparison of median and Bayesian approaches for the estimation of IC_50_ values for inotropy changes.

For validation, the comparison of simulated data with experimental recordings of drug-induced effects on contractility is interpreted in light of the following factors:

(i) quality and completeness of the input data (i.e., the quantitative characterisation of drug-induced modes of action, including ion channel screening and knowledge of additional inotropic mechanisms);
(ii) the *in silico* model used, including equations and parameters (i.e., the population of human cell models based on the Margara2021 electromechanical model);
(iii) simulation protocols applied (including stimulation rate, ionic concentrations, and duration); and
(iv) experimental conditions of *in vitro* data on contractility (i.e., isolated cardiomyocytes affected by isolation procedures; Yue et al., 1996).

## 3 Results

### 3.1 *In silico* predictions of negative inotropic effects quantitatively match *in vitro* observations

An analysis performing pure ion channel block simulations (Supplementary Material) suggested AT_peak_ as the most informative biomarker for predicting drug-induced effects on cardiac inotropy. Simulation results focused on this biomarker show a quantitative agreement with drug-induced neutral/negative inotropic effects observed experimentally for the reference compounds from Dataset 1 (Nguyen et al., 2017).

**FIGURE 3:**
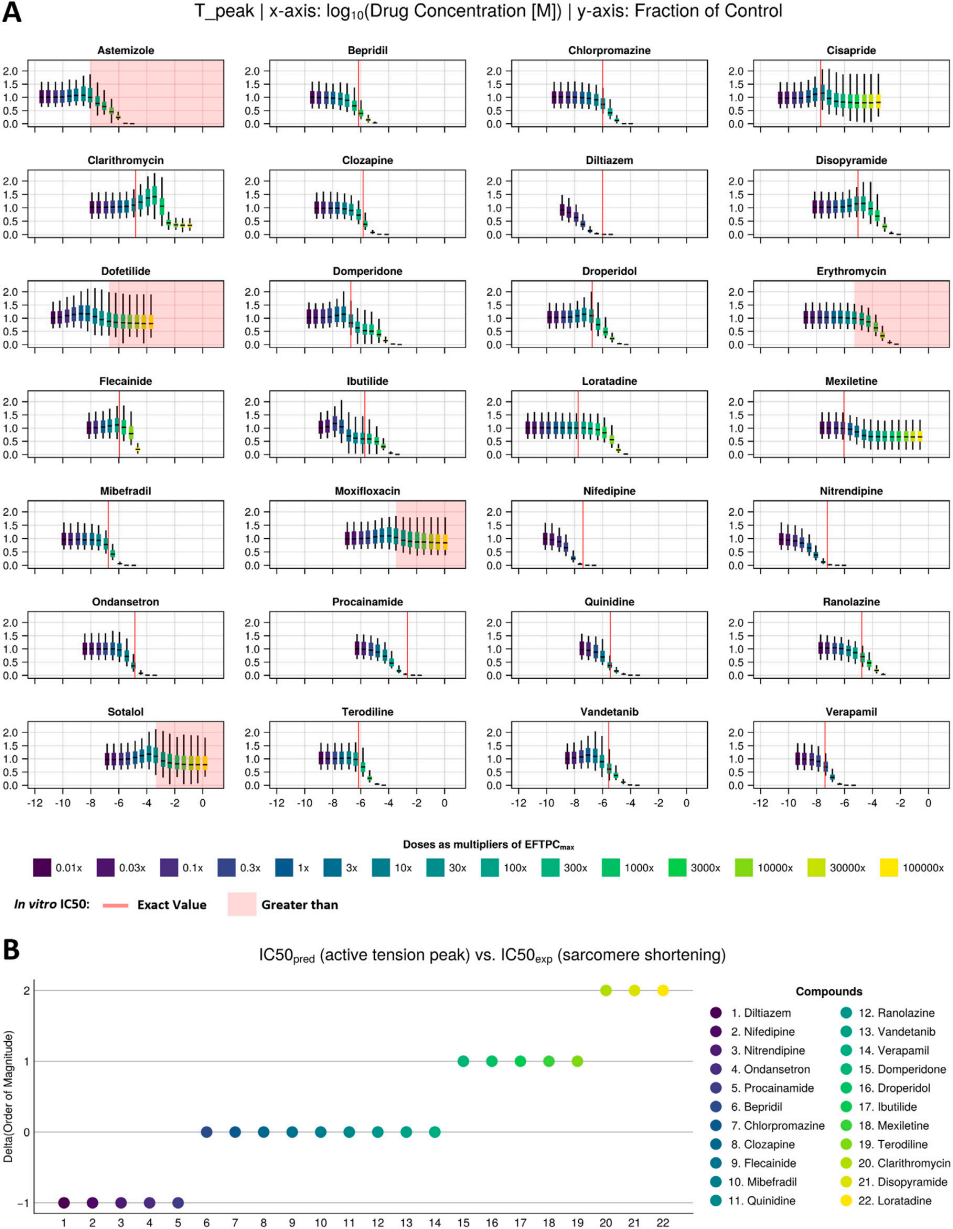
Drug-induced negative inotropic effects for the 22 compounds with variable modes of action. **(A)** Visual comparison between the *in silico* predicted change in peak tension under drug effects at multiple concentrations (sigmoids) and *in vitro* IC_50_ values (red line/area) of sarcomere shortening dose–response curves measured in 3–8 samples from 1 or 2 donor hearts (Nguyen et al., 2017). **(B)** Quantitative comparison between *in silico* and *in vitro* inotropic predictions expressed as the difference between the predicted and experimentally measured IC_50_ values’ order of magnitude. The colour scale from dark blue to yellow refers to dose as a multiplier of EFTPCmax in both panels.

Figure 3A shows predicted drug-induced changes in AT_peak_ for the 28 reference compounds in Dataset 1. Predicted values are shown as fractions of the control value (no drug) and reported for the full population of models in the form of box and whisker plots. To facilitate visual comparison with *in vitro* observations, IC_50_ values obtained from Nguyen et al. (2017) were either plotted as red vertical lines if the associated compounds showed a negative inotropic effect or indicated as a red area if the compounds showed no effect. This approach aligns with the authors’ suggestion that any effect should arise only past the highest tested concentration. On the other hand, to facilitate the comparison with clinical EFTPC_max_, boxplots were coloured based on the ratio between the dose tested and EFTPC_max_, covering large concentration ranges, from 0.01× (dark purple) to 100,000× (yellow).

*In silico* and *in vitro* results were in agreement for 25 out of these 28 compounds, with 22 compounds showing a negative inotropic effect (i.e., a median change from baseline of more than 25%, as detailed by Nguyen et al. (2017) and 3 compounds (dofetilide, moxifloxacin, and sotalol) showing no inotropic effect. Of the remaining three compounds, two (astemizole and erythromycin) showed a negative inotropic effect *in silico* at concentrations above 10× the EFTPC_max_ but not *in vitro*, and one (cisapride) showed a negative inotropic effect *in vitro* but not *in silico*.

For the 22 compounds showing negative inotropic effects both *in vitro* and *in silico*, a quantitative comparison was performed, as discussed below. Table 2 reports both *in vitro* IC_50_ values obtained from Nguyen et al. (2017) and *in silico* IC_50_ values computed from peak tension dose–response curves, fitted using both the median and Bayesian approaches, as described in Section 2.3.3. The ratios of IC_50_ values and clinical EFTPC_max_, which are often used to define safety margins, are reported in brackets. To facilitate the numerical comparison, Figure 3B shows the quantitative difference between the *in silico* and *in vitro* IC_50_ values for the 22 negative inotropic compounds. Each drug is represented as a coloured circle and classified based on the difference between the *in silico* and *in vitro* IC_50_ values’ order of magnitude. We will refer to this quantity as “delta” for simplicity. A delta value of 0 indicates that the predicted IC_50_ value for a given compound had the same order of magnitude as the experimentally measured one. A negative delta value indicates that a smaller IC_50_ value was predicted *in silico* (conservative/worst-case scenario prediction). Finally, a positive delta value indicates that a larger IC_50_ value was predicted (a more risky prediction).

Out of 22 compounds, 19 had a delta value lower than or equal to 1 in absolute value, meaning that the predicted IC_50_ value was either of the same order of magnitude as the experimentally measured one or it was either 10 times larger (mismatch of 1) or smaller (mismatch of −1). Nine compounds (bepridil, chlorpromazine, clozapine, flecainide, mibefradil, quinidine, ranolazine, vandetanib, and verapamil) had a delta value of 0; five compounds (diltiazem, nifedipine, nitrendipine, ondansetron, and procainamide) had a delta value of −1; and five compounds (domperidone, droperidol, ibutilide, mexiletine, and terodiline) had a delta value of 1. The remaining 3 out of 22 compounds (clarithromycin, disopyramide, and loratadine) had a delta value of 2 (100 times larger predicted IC_50_).

The mismatches presented in Figure 3B were calculated from the IC_50_ values derived using the median approach. However, in Section 2.3.3, we also presented a second approach that uses a Bayesian framework to calculate full posterior distributions for the IC_50_ values. Summary statistics (mean and standard deviation) of these distributions are reported in Table 2. When calculating the deltas using the IC_50_ distribution mean values, we obtained consistent results throughout, except from two compounds, namely, flecainide and domperidone, which, this time, were predicted to have a higher delta value (from 0 to 1 and from 1 to 2, respectively).

Regarding safety margins, the comparison between *in vitro* and *in silico* predictions reflects the same trends observed for IC_50_ values. Therefore, for 19 compounds, *in vitro* and *in silico* margins are quantitatively consistent, at least within an order of magnitude, increasing confidence in those predictions and informing dose selection for *in vivo* studies.

### 3.2 Predictions of positive inotropic changes across several modes of action

**FIGURE 4:**
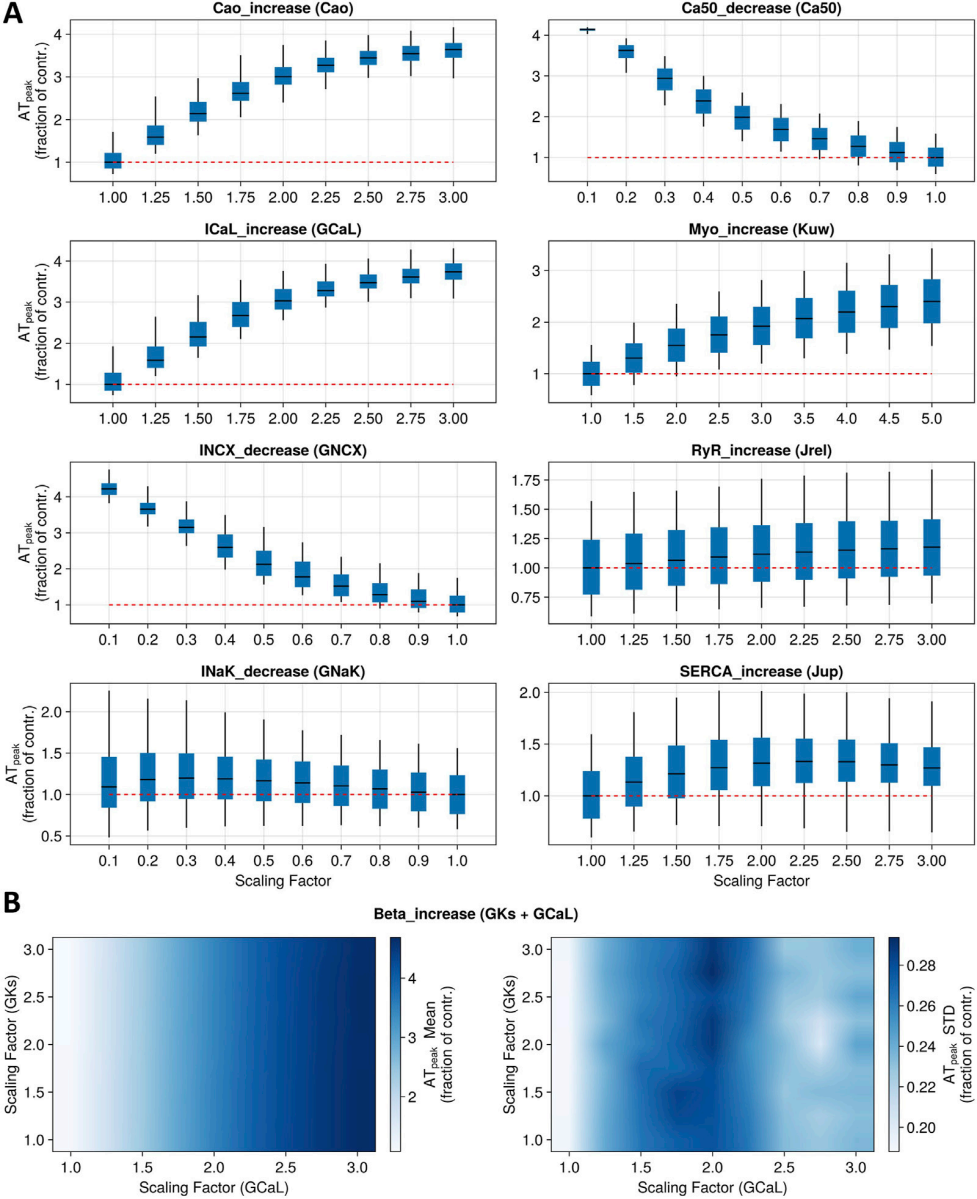
Sensitivity analysis for nine model mechanisms corresponding to main known modes of action of positive inotropic drugs. **(A)** Boxplots report full population variability in peak tension (as a fraction of control) when altering one parameter at a time to represent the first eight mechanisms. Red dotted lines represent the baseline values. **(B)** Heatmaps show population summary statistics (left panel: mean; right panel: standard deviation) for peak tension (as a fraction of control) when altering two parameters simultaneously to represent the ninth mechanism under study (beta-adrenergic stimulation).

Figure 4 shows a summary of the predicted changes in cardiac contractility (peak active tension) induced by the simulation of inotropic mechanisms considered in this study, beyond ion channel inhibition. As described in Section 2.3.4, these mechanisms were selected based on the compounds investigated by Abi-Gerges et al. (2020) in human primary cardiomyocytes (Dataset 2) and evaluation of the readiness of representing those mechanisms in the model. Each panel of Figure 4A reports box and whisker plots for the AT_peak_ fraction of control (model with no perturbed parameters) simulated using the whole population of models, following the modulation of single parameters corresponding to the first eight mechanisms under study. Figure 4B shows summary statistics (mean and standard deviation) of the AT_peak_ fraction of control, following modulation of two parameters corresponding to β-adrenergic stimulation. Selected parameters and specific perturbation ranges used are reported in Table 3.

Simulations of the direct increase in extracellular Ca^2+^ concentration, ranging from 1.8 to 5.4 mM, led to an AT_peak_ value of 362%, where 100% indicates the control condition. The reduction in Ca^2+^ sensitivity, up to 90% of its baseline value, induced an increase in contractility (AT_peak_ = 414%). Similarly, increasing G_CaL_ up to 3-fold its baseline values resulted in an AT_peak_ value of 375%. On the other hand, reducing the permeability of the Na^+^–Ca^2+^ exchanger induced an AT_peak_ value of 452%. Instead, reducing the permeability of I_NaK_ led to a modest increase in contractility (maximum AT_peak_ = 121%). Increasing myosin activation up to 5-fold its baseline value led to an AT_peak_ value of 242%. Increasing the flux of Ca^2+^ through RyR channels up to 3-fold its baseline values had mild effects on the tension peak, leading to a maximum AT_peak_ value of 114%. Similarly, increasing SERCA Ca^2+^ uptake flux led to an ATpeak of 133%. β-adrenergic stimulation was simulated with up to a 3-fold increase in ionic conductances for I_CaL_ and I_Ks_ (Dorian, 2005; Taggart et al., 2021), leading to an increase in peak tension of 450%. However, our simulations show that when altering both conductances at the same time, changes in tension were mainly driven by I_CaL_, while I_Ks_ modulation had little to no contribution (Figure 4B).

A physiologically relevant parametrisation for seven out of nine (all but Na^+^/K^+^ ATPase inhibition and RyR activation) model parameters could be identified that quantitatively recapitulates drug-induced positive inotropic effects for 10 out of 13 reference compounds from Dataset 2 (Abi-Gerges et al., 2020). This means that each parameter could be scaled via a specific factor to simulate an increase in peak tension that matches exactly (as a percentile variation from the control value) experimentally observed sarcomere shortening at the EC_50_ value for those compounds whose main mode of action was represented by the mechanism. Calculated scaling factors and parameters altered for each mode of action are summarised in Table 3.

Extracellular Ca^2+^ modulation was investigated *in vitro* using CaCl_2_, which led to a maximum sarcomere shortening of 220%. As described above and reported in Table 3, simulations covered the range observed experimentally for this mode of action. A scaling factor of 1.24 was computed for the extracellular Ca^2+^ concentration to obtain the same magnitude of change in contractility observed *in vitro* at the EC_50_ value (120%). Levosimendan is a Ca^2+^ sensitizer, which induced a mild increase in sarcomere shortening *in vitro (*131%*)*, smaller than the maximum increase obtained *in silico* (414%). A scaling factor of 0.85 was computed to reproduce the increase in contractility observed *in vitro* at the EC_50_ value (115%).

Bay-K 8644 was used *in vitro* to increase I_CaL_, leading to a maximum increase in contractility of 180% and an increase of 138% at the EC_50_ value, which was obtained *in silico* by applying a scaling factor of 1.17 to I_CaL_ conductance. To inhibit the Na^+^–Ca^2+^ exchanger *in vitro*, Abi-Gerges and others used SEA-0400 and observed a maximum sarcomere shortening of 168%. A scaling factor of 0.79 was computed for G_NCX_ to simulate the same increase in contractility observed at the EC_50_ value. Two compounds were tested *in vitro* as Na^+^/K^+^ ATPase inhibitors, digoxin and ouabain, which led to similar AT_peak_ values of 250% and 237%, respectively. In this case, as described above, simulations were not able to reproduce the same magnitude of change, and the scaling factors to simulate the effects of these two drugs at the EC_50_ value could not be computed.

Two drugs were also tested *in vitro* to investigate myosin activation effects, namely, EMD-57003 and omecamtiv mecarbil, which led to sarcomere shortening of 368% and 263% at the EC_50_ value, respectively. For this mode of action, simulations were able to reproduce the changes observed at the EC_50_ value using 4.53 and 2.63 as scaling factors of myosin activation for EMD-57003 and omecamtiv mecarbil, respectively.

In terms of excitation–contraction coupling mechanisms, caffeine increases calcium-induced-calcium-release through RyR activation, leading to maximum sarcomere shortening *in vitro* of 261%, which was not reached *in silico* as described above. N-106 is a SERCA activator, which induced a maximum AT_peak_ value of 148% *in vitro*. The maximum AT_peak_ obtained via simulations was 133%, enough to compute a scaling factor of 1.62 to mimic the effects observed *in vitro* at the EC_50_ value (124%).

Three β-adrenergic agonists were tested *in vitro*, namely, dobutamine, isoproterenol, and epinephrine, which led to AT_peak_ values of 126%, 334%, and 180%, respectively. As previously described, modulation of I_Ks_ played no role as the cumulative simulated positive inotropic effect was mainly I_CaL_-driven. Therefore, we only reported I_CaL_ scaling factors, amounting to 1.22, 1.56, and 1.30 for reproducing the changes observed *in vitro* at the EC_50_ values for dobutamine, isoproterenol, and epinephrine, respectively.

## 4 Discussion

In this study, we predicted drug-induced effects on cardiac cellular inotropy using multiscale simulations on a population of 323 *in silico* human ventricular healthy cells, and the input included *in vitro* data or assumptions related to drug-induced effects on ionic currents and other mechanisms of inotropy. We considered a set of 41 reference compounds as a validation dataset: 28 drugs inhibiting specific cardiac ion channels, leading to negative or non-inotropic effects, and 13 compounds having heterogeneous modes of action, leading to positive inotropic changes. *In silico* contractility biomarkers were then compared with published *in vitro* data and clinical observations of drug-induced inotropy effects (Gao et al., 2023; Garg et al., 2024; Harmer et al., 2012; Pointon et al., 2015).

The main findings of this study are as follows:

• Simulations of pure ion channel blocks identify the active tension peak as the best surrogate biomarker of sarcomere shortening, with high predictive potential.
• *In silico* simulations using the human electromechanical cell model described by Margara et al. (2021) and *in vitro* ion channel data well-predicted drug-induced neutral/negative inotropic changes for all tested compounds whose main mode of action was ion channel inhibition. For 25 out of 28 compounds, *in silico* predictions were consistent with *in vitro* observations. Of these, 19 out of 22 (86%) also showed quantitative agreement within an order of magnitude.
• Simulations could qualitatively reproduce drug-induced positive inotropic changes for all tested compounds, whose main mode of action corresponded to any of the nine model mechanisms tested. For 10 out of 13 compounds, *in silico* predictions were qualitatively consistent with *in vitro* observations.

Among the compounds from Dataset 1 (negative/neutral inotropic effects), simulations with the Margara2021 model replicated the trend observed *in vitro* for all compounds (Figure 3A), except for cisapride, astemizole, and erythromycin. Cisapride induced negative inotropic effects *in vitro* and predicted no effects *in silico*. However, no clinical reports have highlighted the significant effects of cisapride on cardiac contractility, and concentrations eliciting a decrease in contractility *in vitro* far exceed the anticipated therapeutic exposure. Astemizole, on the other hand, did not induce a negative inotropic effect *in vitro* but it did *in silico* at high concentrations (above 10× the EFTPC_max_), which is consistent with *in vivo* observations of negative inotropic effects in dogs (Sugiyama et al., 1997a; Sugiyama et al., 1997b). Erythromycin had no inotropic effect *in vitro,* but it reduced active tension *in silico* at high concentrations (above 10× the EFTPC_max_). Erythromycin has been associated with arrhythmia and QT prolongation, but no *in vivo* effects on cardiac inotropy have been reported.

*In vitro* and *in silico* contractility IC_50_ values were quantitatively in agreement (within an order of magnitude) for 19 of the investigated compounds (Figure 3B). For quantitative predictions, it is important to highlight that the outcome of simulations strongly depends on the input data representing the drug action. For example, a different set of ion channel data for simulating diltiazem effects was available in the literature (Crumb et al., 2016), leading to a more negative delta value (−2) compared to simulations performed using ion channel data obtained from Kramer et al. (2013). In this study, we used IC_50_ values obtained from Kramer et al. (2013) since they also include diltiazem-induced inhibition of the I_Na_ current, which is significant (Mirams et al., 2011).

For three compounds (clarithromycin, loratadine, and disopyramide), the delta value between *in vitro* and *in silico* IC_50_ values was of two orders of magnitude. *In silico*, clarithromycin induced significant positive inotropic changes at concentrations 10–300 times that of EFTPC_max_ and negative inotropic effects only at extremely high concentrations (∼2000× EFTPC_max_). Therefore, it could not be predicted as a negative inotropic drug. The biphasic behaviour of clarithromycin is due to its multichannel effect: the initial block of hERG leads to a positive inotropic effect as prolonging the AP leads to more Ca^2+^ entering the cell, whereas at higher concentrations, the block of I_Na_ becomes dominant, leading to lower AP amplitude and plateau and, therefore, to less Ca^2+^ influx and reduced inotropy. The ratio between the *in vitro* IC_50_ value and EFTPC_max_ was 13× (Table 2), despite no clinical observations suggesting negative inotropic potential for clarithromycin (Gluud et al., 2008; Suzuki et al., 2012; Wong et al., 2016). The effects of clarithromycin on the cardiovascular system are not clear and still debated. Clarithromycin has been associated in the short term with increased risks of myocardial infarction, prolonged QT, arrhythmia, and cardiac mortality (Wong et al., 2016). In the long term, clarithromycin increased 6-year mortality in coronary heart disease patients (Gluud et al., 2008), but its inotropic effects have not been assessed.

Similarly, simulations for loratadine show negative inotropic effects only at concentrations thousands of times that of EFTPC_max_, consistent with the cardiac safety profile of this drug (Hey et al., 1999; Nault et al., 2002). Disopyramide induced mild positive inotropic changes at concentrations 10–100 times that of EFTPC_max_ and negative inotropic effects only at higher concentrations, leading to an *in silico* IC_50_ value of ∼400 μM, higher than *in vitro* observations in human (Nguyen et al., 2017) and canine (Harmer et al., 2012) cardiomyocytes (9.3 µM and 44 μM, respectively). *In vivo* and clinical evidence highlighted the significant effects of disopyramide on cardiac contractility (Coppini et al., 2019; Kim and Benowitz, 1990; Pollick et al., 1982), suggesting that in addition to I_CaL_ inhibition, disopyramide might affect contractility via other modes of action.

Regarding safety margins, there is no official threshold provided by regulators for cardiac contractility assessment, although 100× EFTPC_max_ (or 100× of the maximum free plasma concentration of a drug) is usually considered a robust range to explore. *In silico* predictions of contractility changes were quantitatively consistent (within an order of magnitude) with *in vitro* observations for 86% of negative/neutral inotropic compounds from Dataset 1 (Table 2).

The slightly worse performance of the Bayesian point estimates in this study should not overshadow its broader utility. When using the Bayesian method, posterior distributions highlight how uncertainty might contribute to discrepancies, offering insights that a median fit cannot provide. For example, wider uncertainty ranges observed for a compound could signal experimental or model-specific variability worth further investigation. Although the median fit approach offers simplicity and computational ease, it is inherently limited in its ability to handle variability, which is a hallmark of biological systems. The Bayesian framework, although more computationally intensive, provides a more comprehensive characterisation of uncertainty and is, therefore, better suited for general application, especially when variability is significant. We consider that the apparent superiority of the median fit in this specific dataset is circumstantial and does not detract from the overall advantages of the Bayesian approach.

Considering the inotropic effect of ion channel inhibition alone only accounts for one specific mechanism of toxicity (mostly calcium reduction), and therefore, any effects via other mechanisms would not be predicted. Beyond ion channel inhibition, perturbation of other inotropic mechanisms such as myosin activation or calcium sensitivity could affect cardiac contractility. Simulations with the Margara2021 model reproduced the drug-induced effects observed *in vitro* (Table 3) for the following mechanisms: (1) extracellular Ca^2+^ concentration increase; (2) I_CaL_ activation; (3) SERCA pump activation; (4) Na^+^/Ca^2+^ exchanger inactivation; (5) β-adrenergic stimulation; (6) Ca^2+^ sensitivity decrease; and (7) cardiac myosin activation. For Na^+^/K^+^ pump inactivation and RyR activation, simulations are qualitatively consistent with *in vitro* observation but do not replicate the range observed *in vitro*, due to the modelling approach chosen to describe those two modes of action. These results were obtained after perturbating mostly only one selected parameter for each mode of action. In addition, omecamtiv mecarbil, a myosin-activating drug, was simulated perturbating only one selected parameter following the approach described by Tewari et al. (2016), i.e., assuming that the drug increases the rate of myosin head binding with the actin filament. Specifically, Tewari and others simulated the omecamtiv mecarbil mechanism of action by increasing their model parameter k_a_, representing the rate of myosin-head attachment with actin. In our specific case [cell contraction model from Land et al. (2017)], this was translated into scaling the K_uw_ parameter, representing the transition rate from a cross-bridge unbound state to a cross-bridge weakly bound state. However, Kampourakis et al. (2018) describe omecamtiv mecarbil as exhibiting a biphasic behaviour, which was not captured in our simulations, but it might be reproduced using more sophisticated modelling and simulation approaches (Campbell et al., 2018; Forouzandehmehr et al., 2022). Overall, the rationale behind our modelling approach was not to provide a detailed description of each drug’s mode of action, but, instead, to use the simplest modelling approach that could be more easily characterised and informed using early *in vitro* data during the drug development process. Future studies could explore more complex approaches to model these modes of action and identify datasets to quantitatively understand drug effects on these mechanisms (Terkildsen et al., 2007). The adoption of more sophisticated models to describe cardiac mechanical contraction might also affect our finding that the AT peak is the most informative biomarker for assessing drug-induced effects on cardiac contractility. A systematic analytic comparison of contractility biomarkers across several mechanical models, however, goes beyond the scope of the present study. As a future work, additional modes of action and/or biological mechanisms (e.g., ATP hydrolysis) could also be implemented into the Margara et al. (2021) model to expand the applicability of the proposed modelling framework, including its use in studying disease-related/drug-induced metabolic impairments.

As data from experimental assays quantifying the extent of perturbation of these nine contractility mechanisms were not available, a sensitivity analysis approach was adopted. This aimed to gain an understanding of the extent of predicted changes in cardiac contractility biomarkers *in silico* under different degrees of perturbation for each of these mechanisms. When information on the potential involvement of a particular mechanism is available, which may not be fully quantitative (e.g., predicted from the compound structure), it can still be leveraged for the early prediction of its impact on cardiac inotropy, either alone or in combination with other mechanisms (e.g., ion channel inhibition). If an IC_50_ value for a given mechanism is known, it could be incorporated into simulations for a more quantitative prediction of the expected effect on cardiac contractility. Otherwise, a range of modulations can be explored to compute an early therapeutic index and inform dosing strategies for *in vivo*/clinical studies. The code provided allows for the simulation of any of these mechanisms, alone or alongside ion channel inhibition, by varying mechanism-specific model parameters. This enables predictions for both positive and negative inotropic compounds.

To move toward quantitative predictions for positive inotropic compounds, additional preclinical data are needed to characterise each of the abovementioned mechanisms for positive inotropy in a dose-dependent way. Similar to the CiPA initiative, a newly conceived, hypothetical initiative could aim at identifying and accurately characterising reference compounds affecting cardiac inotropy to define *in vitro/in silico* approaches and dose-dependent input data for modelling and simulations, similar to IC_50_ values for describing ion channel inhibition and predicting arrhythmic risk. This ambitious initiative could only arise from and will require a community effort between academics, pharmaceutical companies, and regulators and is, therefore, beyond the scope of the present study. Moreover, clinical data on drug-induced changes on cardiac contractility for a set of reference compounds will also facilitate the validation of new *in vitro/in silico* assays.

On a final note, cardiac drugs and pathologies can potentially have deleterious effects propagating from the cellular level to the organ scale, which may cause contractile dysfunction and/or increase arrhythmic risk. To capture this complex behaviour, the Margara2021 model can be incorporated into tissue and organ-scale frameworks (Zhou et al., 2024; Margara et al., 2022), including representations of electrophysiology, electromechanical coupling, and anatomical effects to further improve predictive power and assess mechanistic pathways involved.

## 5 Conclusion

This study describes the validation and application of simulations with an *in silico* human cardiac electromechanical model integrating ion channel inhibition data and information on potential inotropic mechanisms for predicting primary or off-target effects on cardiac contractility. The *in silico* approach was found to predict well inotropy changes in both positive and negative/no effect inotropes. The outlined *in silico* modelling and simulation workflows (Figure 1A) could inform how this model can be used as part of cardiac safety assessment strategies, along with computational models for predicting drug-induced changes in cardiac electrophysiology within pharmaceutical research and development.

## Data Availability

All data and code used for running drug-block and mechanism-perturbation model simulations, dose/perturbation-response curves’ fitting, and plotting are available on a GitHub repository as a Julia package at https://github.com/GSK-Biostatistics/CellContraction.jl.

## Abbreviations

AP(s), action potential(s); APA, action potential amplitude; APD_x_, AP duration at X% of repolarisation; AT, active tension; Ca^2+^, calcium [Ca^2+^]_i_, intracellular Ca^2+^ concentration; CaT, Ca^2+^ transient; BCL, basic cycle length; dV/dt_MAX_, maximum upstroke velocity; EAD(s), early after-depolarisation(s); EMw: electromechanical window; EOP, membrane potential at the end of repolarisation; G_X_, I_X_ conductance; IC_50_, drug concentration for 50% channel inhibition; I_CaL_, L-type Ca^2+^ current; I_K1_, inward rectifier K^+^ current; I_Kr_, rapid delayed rectifier K^+^ current; I_Ks_, slow delayed rectifier K^+^ current; I_Na_, fast Na^+^ current; I_NaK_, Na^+^/K^+^ pump current; I_NaL_, late Na^+^ current; I_NCX_, Na^+^/Ca^2+^ exchanger current; I_to_, transient outward K^+^ current; K^+^, potassium; MoA, mode of action; Na^+^, sodium; SS, steady state; V_m_, membrane potential.
