# Supplementary material for: In silico predictions of drug-induced changes in human cardiac contractility align with experimental recordings
Source: Front Pharmacol. 2025 Mar 17;16:1500668. doi: 10.3389/fphar.2025.1500668 (PMC11955705; doi:10.3389/fphar.2025.1500668)
Supplement: Supplementary file 1 [file DataSheet1.pdf]

In silico predictions of drug-induced changes in human cardiac contractility align with experimental recordings

SUPPLEMENTAL MATERIAL

Contents

Effects of simulated pure blockers on cardiac contractility....................2
Supplementary Figures....................3
Figure S1. Model code verification....................4
Figure S2. Biomarker changes induced by ICaL block....................5
Figure S3. Biomarker changes induced by IKr block....................6
Figure S4. Biomarker changes induced by INa block....................7
Figure S5. Percental sarcomere shortening for 13 positive inotropic compounds…………8
Figure S6. Posterior distributions for Ranolazine, Dofetilide, and Verapamil……………..9
Supplementary Table....................
Table S1. Ion channel data for 28 neutral/negative-inotropic compounds....................10
Table S2. Calibration data for population of models....................11
Table S3. Fitted parameter distributions' summary……………………………...…..11
Additional references....................12

Effects of simulated pure blockers on cardiac contractility

The results from the sensitivity analysis for pure I_{CaL}, I_{Kr} and I_{Na} blockers are shown in Figures S2, S3, and S4, respectively. For each channel, current blocks were simulated from 0% (control) to 100% (full block). 18 biomarkers were computed for the whole population of models for each current block and reported as mean and standard deviation.

As expected, I_{CaL} blocks led to a block-dependent reduction of CaT peak (Figure S2G), CaT duration (Figures S2H-I), and diastolic Ca^{2+} concentration (Figure S2F). Reduction of Ca^{2+} concentration led in turn to a reduction of AT: AT peak was reduced up to full suppression (Figure S2K), like AT relaxation time (Figures S2L-M), and maximum and minimum AT_{ttp}, i.e., AT rising velocity (Figures S2N-O). Overall, predicted changes in AT_{peak} are larger than the corresponding predicted changes in CaT_{peak}. I_{CaL} blocks also led to changes in AP morphology: APD_{40} was reduced as well as APD_{90}. Minor changes were observed for RMP, V_{peak} and dV/dt_{max}, as expected. Minor changes were observed for EMw, except at 100% I_{CaL} block, which strongly decreases EMw due to the full suppression of CaT. Tri90-40 increases for I_{CaL} block bigger than 70%, and qNet showed a direct block-dependent increase.

I_{Kr} blocks (Figure S3) increased both APD_{40} and APD_{90}, with minor changes on RMP, V_{peak} and dV/dt_{max}. The main effect observed on CaT is prolonged CTD_{90}, directly related to bigger AP duration. This AP prolongation also led to a small increase in CaT_{peak} up to 70% current block, then, CaT decreases, due to I_{NCX} compensation. AT_{peak} increases for low I_{Kr} and then decreases, following the same trend of CaT.

The main effect of blocking I_{Na} (Figure S4) is a direct block-dependent reduction of dV/dt_{max} up to full suppression. V_{peak} is reduced until 50% current block, then reaches a plateau since it is hold up also by Ca^{2+}. A small decrease was observed for APD_{90}, whereas, minor changes were shown for RMP and APD_{40}. As, expected effects on both CaT and AT were minor. Only I_{Na} blocks higher than 90% led to a strong reduction in CaT and AT, due to a change in the morphology of the AP, which in this case is Ca-driven rather than Na-driven.

Interestingly, standard deviation increases at higher current blocks, showing that *in silico* models start behaviour differently, i.e., their response to strong current block depends on the whole electrophysiological profile of the cell due to the interplay between different ionic currents. This suggests that a comprehensive *in vitro* characterisation of drug-induced effects on cardiac ion channels could improve predictions of cellular response to therapies, despite such characterisation might represent an additional cost during the early drug development process.

It is worth noting that several AT biomarkers were considered (e.g., AT_{ttp} or AT rate of rise/relaxation) for pure ion blockers, but they were excluded from the following analysis as they were similar or less informative than the AT_{peak} biomarker.

Supplementary Figures

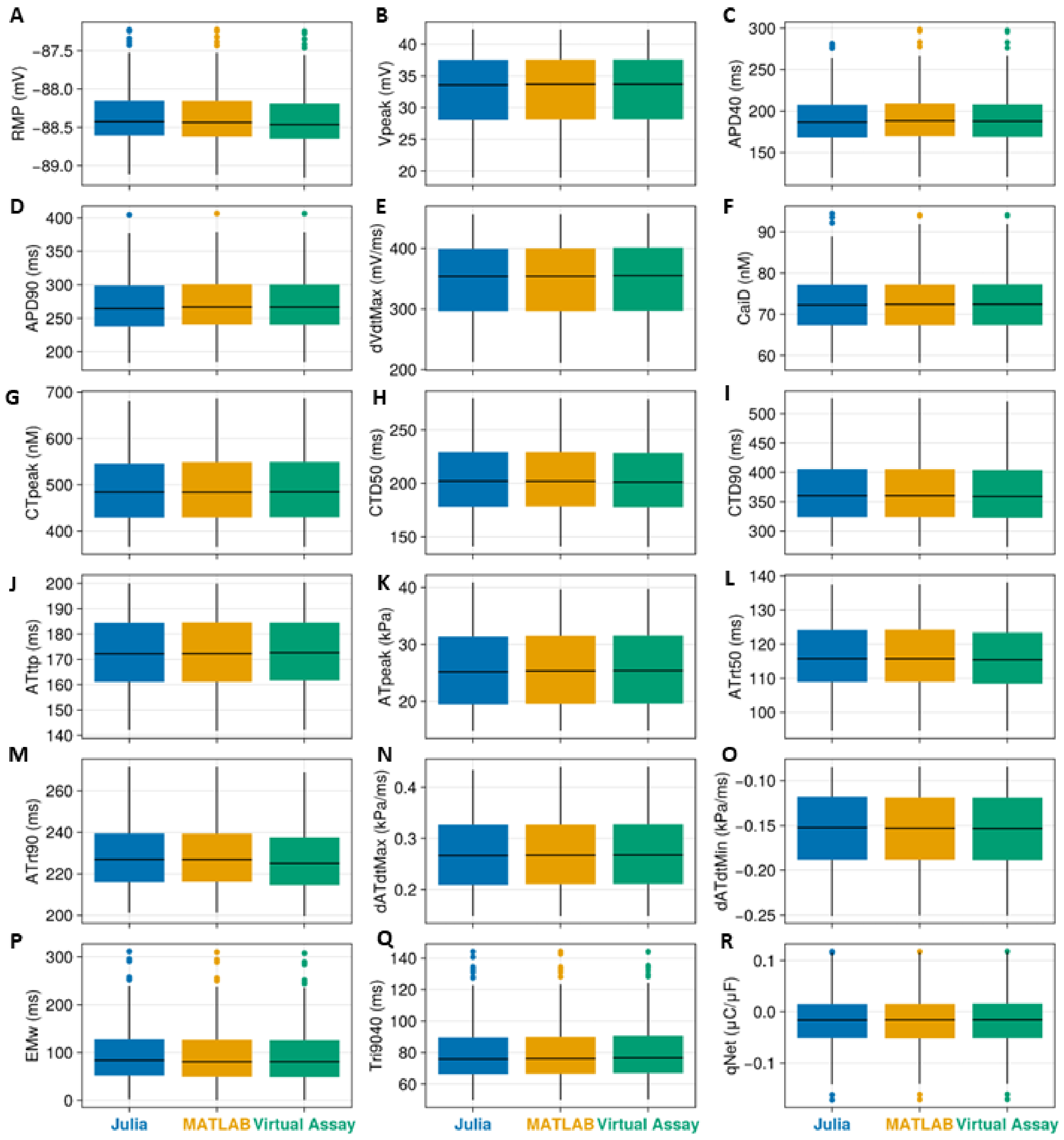

Figure S1. Model code verification. Comparison of the main biomarkers computed using three different coding languages and environments: Julia (blue box), MATLAB (MathWorks®, yellow box), Virtual Assay (green box). A) RMP (mV): resting membrane potential; B) V_{peak} (mV): voltage action potential (AP) peak; C) APD40 (ms): AP duration at 40% of repolarisation; D) APD90 (ms): AP duration at 90% of repolarisation; E) dV/dt_{max} (mV/ms): maximum depolarisation velocity; F) CaiD (nM): intracellular Ca^{2+} diastolic concentration; G) CT_{peak} (nM): Ca^{2+} transient peak; H) CTD50 (ms): Ca^{2+} transient duration at 50% of recovery from peak concentration; I) CTD90 (ms): Ca^{2+} transient duration at 90% of recovery from peak concentration; J) ATttp (ms): time to active tension (AT) peak; K) AT_{peak} (kPa): peak of AT; M) ATrt50 (ms): AT duration at 50% recovery from peak tension; L) ATrt90 (ms): AT duration at 90% recovery from peak tension; M) dAT/dt_{min} (kPa/ms): maximum rate of AT decay; N) dAT/dt_{max} (kPa/ms): maximum rate of AT rise; P) EMw (ms): electromechanical window (EMw = CAD90 - APD90); Q) Tri90-40 (ms): triangulation (Tri90-40 = APD90 - APD40); R) qNet (μC/μF): net cellular charge.

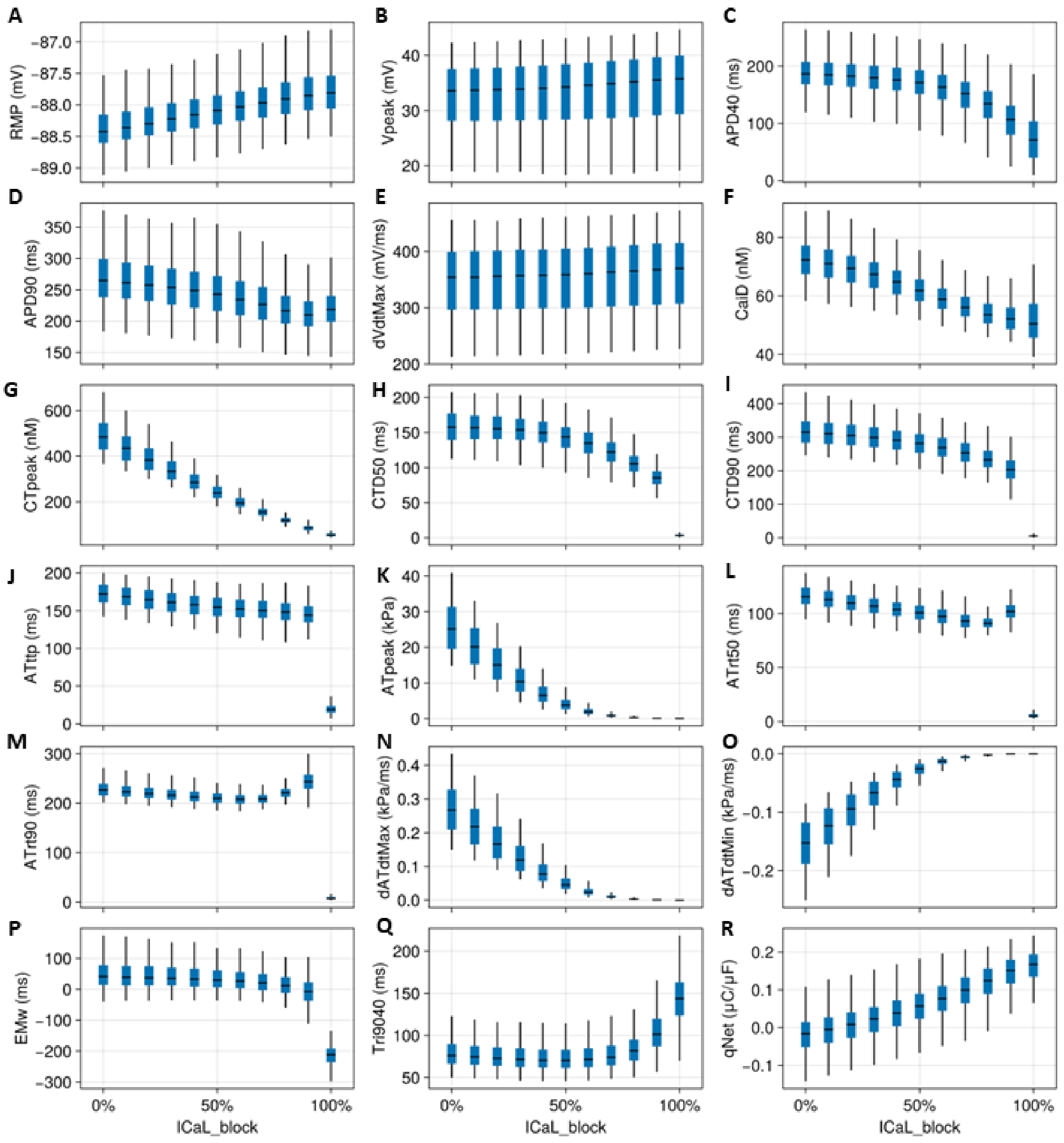

Figure S2. Biomarker changes induced by ICaL block. Biomarker values as calculated from full population of model simulations. A) RMP (mV): resting membrane potential; B) V_{peak} (mV): voltage action potential (AP) peak; C) APD40 (ms): AP duration at 40% of repolarisation; D) APD90 (ms): AP duration at 90% of repolarisation; E) dV/dt_{max} (mV/ms): maximum depolarisation velocity; F) CaiD (nM): intracellular Ca^{2+} diastolic concentration; G) CT_{peak} (nM): Ca^{2+} transient peak; H) CTD50 (ms): Ca^{2+} transient duration at 50% of recovery from peak concentration; I) CTD90 (ms): Ca^{2+} transient duration at 90% of recovery from peak concentration; J) ATttp (ms): time to active tension (AT) peak; K) AT_{peak} (kPa): peak of AT; M) ATrt50 (ms): AT duration at 50% recovery from peak tension; L) ATrt90 (ms): AT duration at 90% recovery from peak tension; M) dAT/dt_{min} (kPa/ms): maximum rate of AT decay; N) dAT/dt_{max} (kPa/ms): maximum rate of AT rise; P) EMw (ms): electromechanical window (EMw = CAD90 - APD90); Q) Tri90-40 (ms): triangulation (Tri90-40 = APD90 - APD40); R) qNet (μC/μF): net cellular charge.

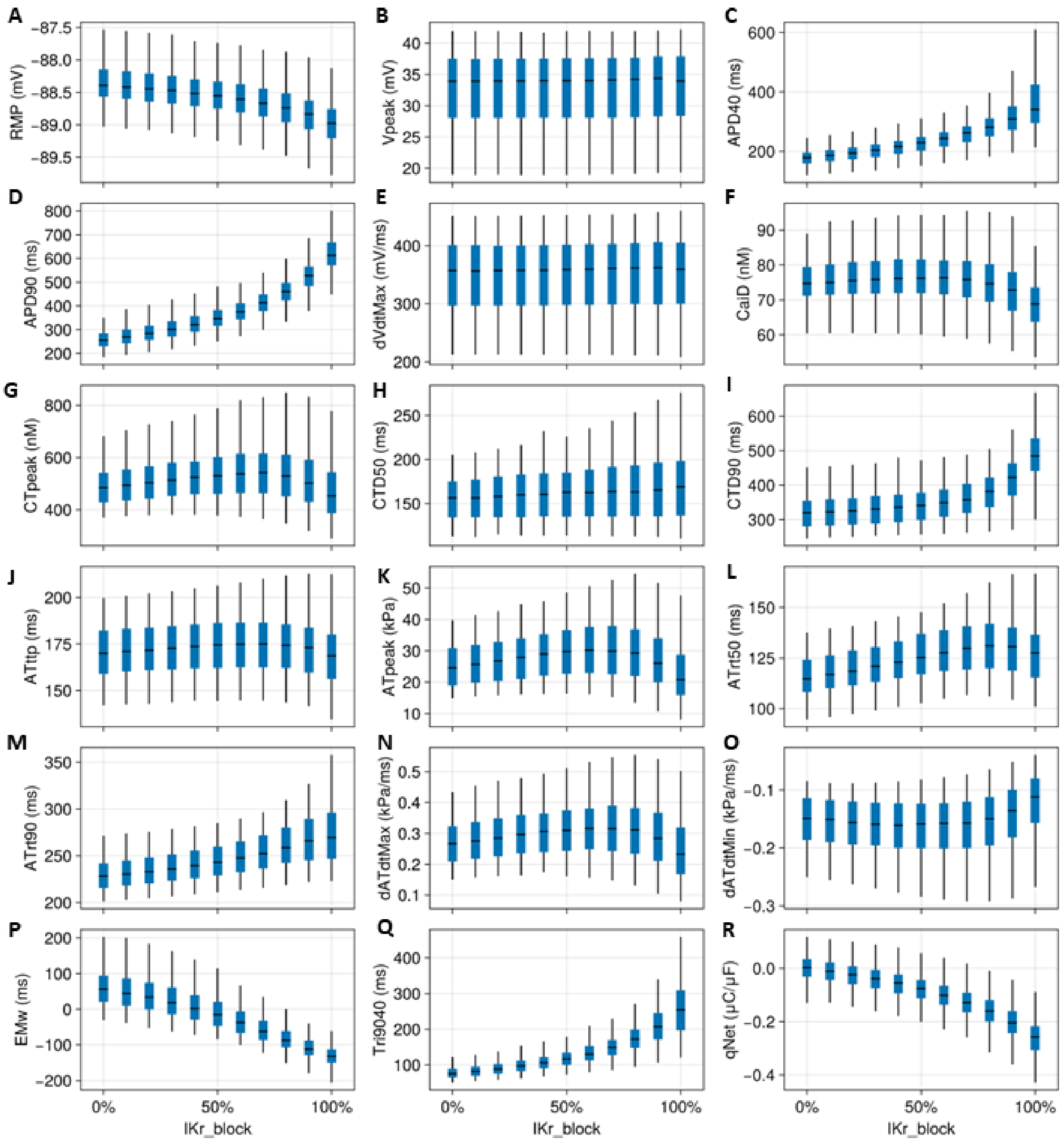

Figure S3. Biomarker changes induced by IKr block. Biomarker values as calculated from full population of model simulations. A) RMP (mV): resting membrane potential; B) V_{peak} (mV): voltage action potential (AP) peak; C) APD40 (ms): AP duration at 40% of repolarisation; D) APD90 (ms): AP duration at 90% of repolarisation; E) dV/dt_{max} (mV/ms): maximum depolarisation velocity; F) CaiD (nM): intracellular Ca^{2+} diastolic concentration; G) CT_{peak} (nM): Ca^{2+} transient peak; H) CTD50 (ms): Ca^{2+} transient duration at 50% of recovery from peak concentration; I) CTD90 (ms): Ca^{2+} transient duration at 90% of recovery from peak concentration; J) ATttp (ms): time to active tension (AT) peak; K) AT_{peak} (kPa): peak of AT; M) ATrt50 (ms): AT duration at 50% recovery from peak tension; L) ATrt90 (ms): AT duration at 90% recovery from peak tension; M) dAT/dt_{min} (kPa/ms): maximum rate of AT decay; N) dAT/dt_{max} (kPa/ms): maximum rate of AT rise; P) EMw (ms): electromechanical window (EMw = CAD90 - APD90); Q) Tri90-40 (ms): triangulation (Tri90-40 = APD90 - APD40); R) qNet (μC/μF): net cellular charge.

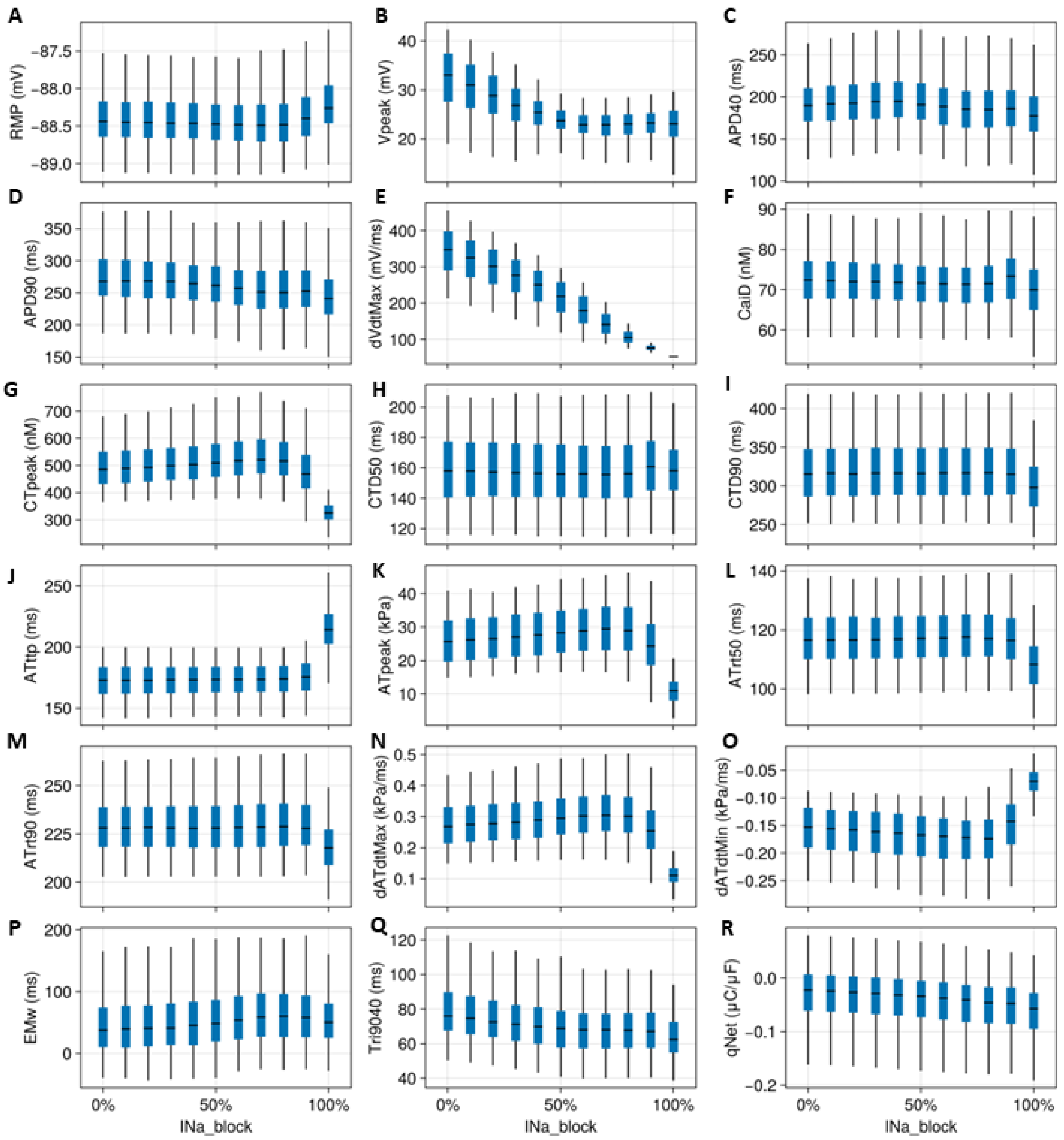

Figure S4. Biomarker changes induced by INa block. Biomarker values as calculated from full population of model simulations. A) RMP (mV): resting membrane potential; B) V_{peak} (mV): voltage action potential (AP) peak; C) APD40 (ms): AP duration at 40% of repolarisation; D) APD90 (ms): AP duration at 90% of repolarisation; E) dV/dt_{max} (mV/ms): maximum depolarisation velocity; F) CaiD (nM): intracellular Ca^{2+} diastolic concentration; G) CT_{peak} (nM): Ca^{2+} transient peak; H) CTD50 (ms): Ca^{2+} transient duration at 50% of recovery from peak concentration; I) CTD90 (ms): Ca^{2+} transient duration at 90% of recovery from peak concentration; J) ATttp (ms): time to active tension (AT) peak; K) AT_{peak} (kPa): peak of AT; M) ATrt50 (ms): AT duration at 50% recovery from peak tension; L) ATrt90 (ms): AT duration at 90% recovery from peak tension; M) dAT/dt_{min} (kPa/ms): maximum rate of AT decay; N) dAT/dt_{max} (kPa/ms): maximum rate of AT rise; P) EMw (ms): electromechanical window (EMw = CAD90 - APD90); Q) Tri90-40 (ms): triangulation (Tri90-40 = APD90 - APD40); R) qNet (µC/µF): net cellular charge.

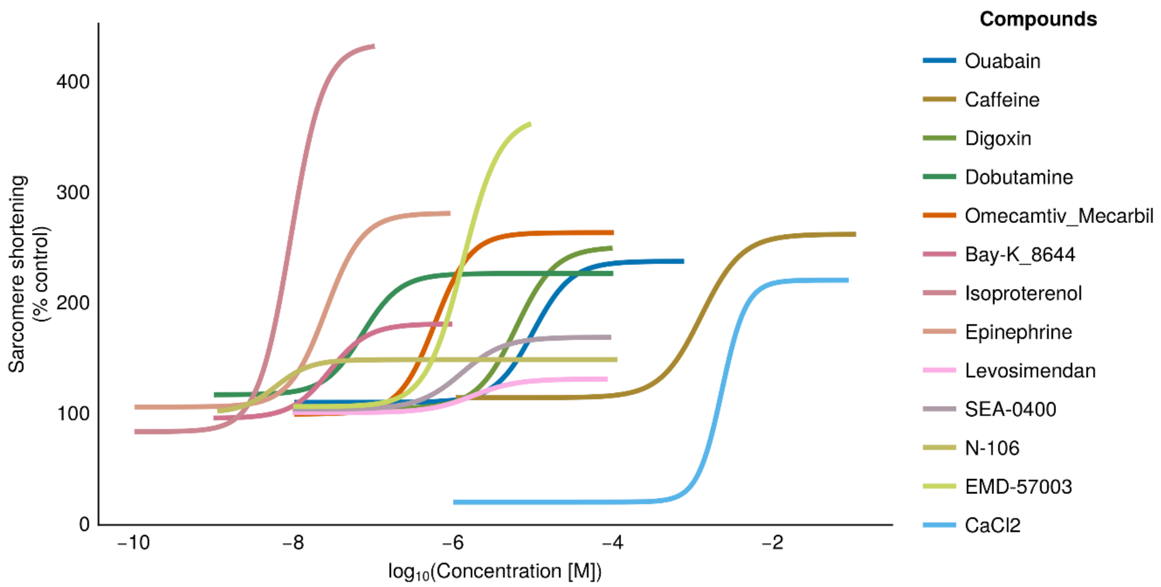

Figure S5. ***In vitro*** **percental sarcomere shortening for 13 positive inotropic compounds.** *In vitro* dose-response curves were recorded from human primary adult cardiomyocytes (Abi-Gerges et al. 2020). Each trace was digitised from its corresponding figure in (Abi-Gerges et al. 2020) and then re-plotted here alongside the others.

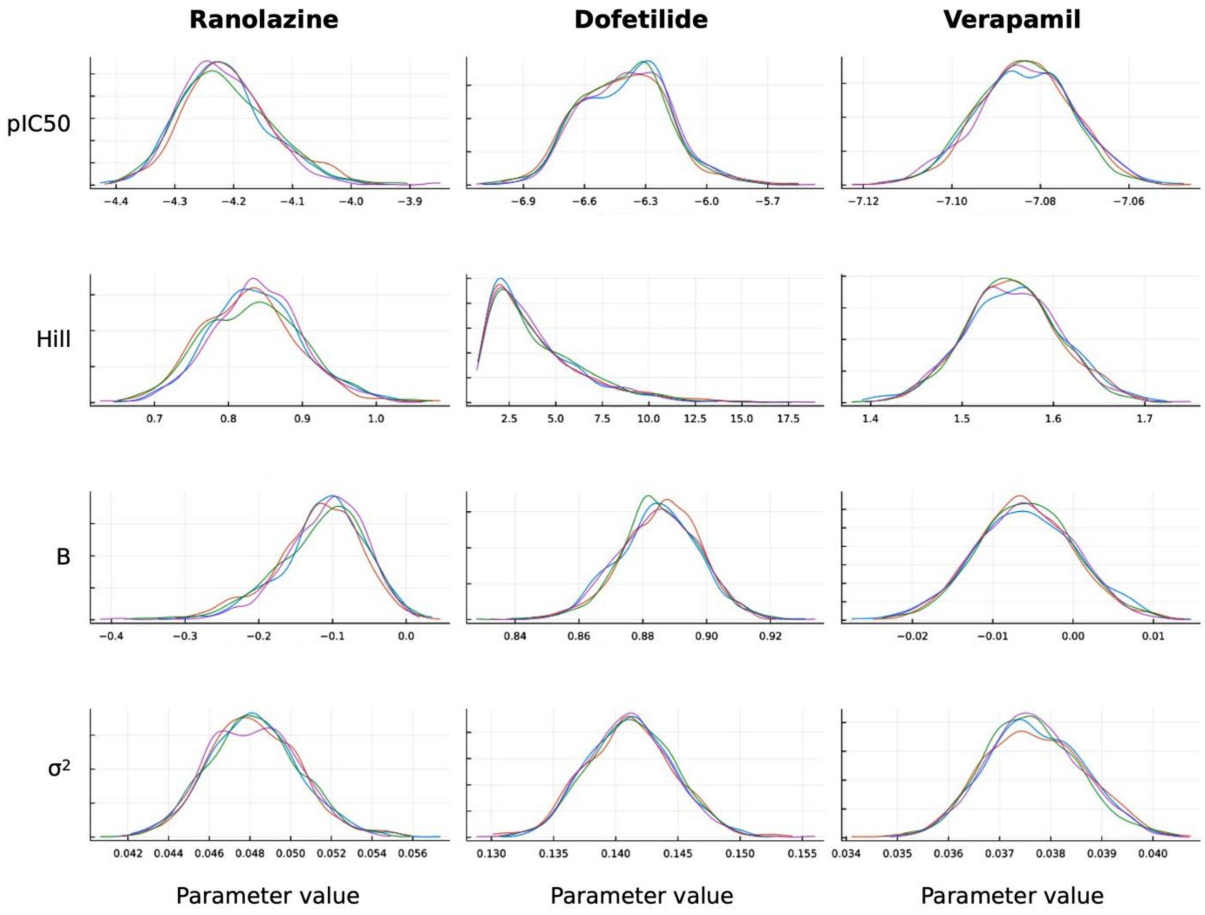

Figure S6. Example one-dimensional views of the posterior distributions for 3 simulated compounds: Ranolazine, Dofetilide, and Verapamil. Different colours represent different Markov chains.

Supplementary Table

Table S1. Ion channel data for 28 neutral/negative-inotropic compounds. Compounds, half-maximal inhibitory concentrations (IC_{50}) and Hill Coefficients (h) for different ion channels, and effective free therapeutic plasma concentrations ($EFTPC_{max}$) used as inputs to perform the *in silico* study. Ion channel data sources were heterogeneous, so for each compound the respective data source is also referenced in the last column.

Compound	GNa		GKr		GCaL		GNaL		GKs		Gto		GK1		EFTPC max	Ref
	h	IC_{50}	h	IC_{50}	h	IC_{50}	h	IC_{50}	h	IC_{50}	h	IC_{50}	h	IC_{50}		
Astemizole	1.95	3	0.78	0.004	1.66	1.1									0.0003	Kramer et al., 2013
Bepridil	1.2	2.929	0.9	0.149	0.6	2.808	1.4	1.814							0.035	Crumb et al., 2016
Chlorpromazine	2	4.536	0.9	1.118	0.8	8.192	0.9	4.56					0.7	9.27	0.0345	Crumb et al., 2016
Cisapride			1.3	0.0123											0.0026	Crumb et al., 2016
Clarithromycin	1	163.576	0.84	62.5											1.2	Passini et al., 2019
Clozapine	1.14	15.1	0.97	2.3	1	3.6									0.071	Kramer et al., 2013
Diltiazem	1.29	22.4	1.16	13.2	1.14	0.76									0.122	Kramer et al., 2013
Disopyramide	1.09	168.4	0.91	14.4	1	1036.7									0.742	Kramer et al., 2013
Dofetilide			0.6	0.001											0.002	Crumb et al., 2016
Domperidone	1	6.322	1.27	0.046	1	50									0.02	Passini et al., 2019
Droperidol	1	1.819	1.2	0.08	1	4.889									0.016	Passini et al., 2019
Erythromycin	1.05	393			0.83	856									0.17	Delaunois et al., 2021
Flecainide	1.9	6.677	0.8	0.692	1.4	25.599	0.6	18.87			0.7	9.266			0.753	Crumb et al., 2016
Ibutilide	1.03	42.5	1.53	0.018	1.16	62.5									0.14	Kramer et al., 2013
Loratadine	1	4.722	1.7	6.2	1	10.21			4.6	10.2					0.00045	Passini et al., 2019
Mexiletine							1.4	8.957							2.5	Crumb et al., 2016
Mibefradil	1	5.866	0.9	0.307	1.1	0.652	1.2	3.628							0.012	Crumb et al., 2016
Moxifloxacin			0.6	93.041			1.1	382.337	1	50.321					10.96	Crumb et al., 2016
Nifedipine	0.71	88.5	0.8	44	1.02	0.012									0.008	Kramer et al., 2013
Nitrendipine	1.25	21.6	0.82	24.6	0.78	0.025									0.003	Kramer et al., 2013
Ondansetron			1	1.492	0.8	22.551	1	19.181							0.372	Crumb et al., 2016
Procainamide	1	746.6	1	272.4	0.83	389.5									54.186	Kramer et al., 2013
Quinidine	1.22	14.6	1.06	0.72	0.68	6.4									3.237	Kramer et al., 2013
Ranolazine	0.8	30.2	0.9	10.9	0.6	172	1	5.9							1.95	Passini et al., 2016
Sotalol			0.9	86.369											14.69	Crumb et al., 2016
Terodiline	1.23	7.4	1.02	0.65	1.01	4.8									0.145	Kramer et al., 2013
Vandetanib	1	53.571	0.9	0.123	1	19.72			1	20					0.3	Passini et al., 2019
Verapamil			1.1	0.499	1.1	0.202									0.045	Crumb et al., 2016

Table S2. Calibration data for population of models. AP, CaT and AT experimental biomarker ranges used to calibrate the population of human ventricular cell electro-mechanical models (Margara et al., 2021; Passini et al., 2019).

AP Biomarkers	Min	Max	CT Biomarkers	Min	Max	AT Biomarkers	Min	Max
APD_{40} (ms)	85	320	CTD_{50} (ms)	120	420	AT_{peak} (kPa)	15	40
APD_{50} (ms)	110	350	CTD_{90} (ms)	220	785	ATttp (ms)	120	200
APD_{90} (ms)	180	440	CaiD (nM)	0	400	ATrt50 (ms)	90	140
Tri90-40 (ms)	50	150	CT_{peak} (nM)	200	1000	ATrt90 (ms)	200	600
dV/dt_{MAX} (V/s)	100	1000						
V_{peak} (mV)	10	55						

Table S3. Fitted parameter distributions' summary statistics (Bayesian approach)

	IC50		Hill		B		sigma2	
**Compound**	**Mean**	**STD**	**Mean**	**STD**	**Mean**	**STD**	**Mean**	**STD**
Astemizole	0.3096	0.0487	1.0322	0.1014	-0.0401	0.0425	0.0703	0.0037
Bepridil	0.8400	0.0647	1.0408	0.0558	-0.0347	0.0254	0.0573	0.0015
Chlorpromazine	2.9157	0.1347	1.2746	0.0625	-0.0180	0.0112	0.0504	0.0015
Cisapride	0.2339	0.0734	5.4551	2.5922	0.8778	0.0100	0.1245	0.0032
Clarithromycin	2795.6898	250.9413	7.7708	2.2463	0.3520	0.0194	0.1125	0.0038
Clozapine	1.6234	0.0697	1.4291	0.0772	-0.0128	0.0125	0.0488	0.0016
Diltiazem	0.3019	0.0077	1.6290	0.0580	-0.0071	0.0067	0.0399	0.0010
Disopyramide	468.2128	26.6650	1.7890	0.1096	0.0015	0.0195	0.0803	0.0025
Dofetilide	0.4494	0.2590	3.9432	2.5654	0.8852	0.0126	0.1412	0.0035
Domperidone	10.9122	2.3777	0.6354	0.0414	-0.0919	0.0436	0.0951	0.0036
Droperidol	1.8256	0.1264	1.4801	0.0975	0.0078	0.0203	0.0723	0.0025
Erythromycin	318.0348	20.8735	1.1843	0.0673	-0.0340	0.0216	0.0609	0.0016
Flecainide	10.3227	1.6724	5.6753	2.2118	0.1647	0.0794	0.0886	0.0025
Ibutilide	27.7873	9.7651	0.4510	0.0245	-0.2901	0.0782	0.0953	0.0029
Loratadine	5.7290	0.3355	1.3389	0.0771	-0.0535	0.0247	0.0663	0.0014
Mexiletine	9.9173	1.0542	1.7553	0.2861	0.6962	0.0052	0.0665	0.0014
Mibefradil	0.3021	0.0141	1.6685	0.1190	-0.0071	0.0125	0.0467	0.0019
Moxifloxacin	9715.5457	7097.2535	3.7916	2.5531	0.9061	0.0124	0.1133	0.0025
Nifedipine	0.0041	0.0001	1.4565	0.0548	-0.0064	0.0075	0.0309	0.0011
Nitrendipine	0.0060	0.0003	1.0505	0.0453	-0.0123	0.0093	0.0320	0.0012
Ondansetron	8.0179	0.2416	1.5501	0.0594	-0.0006	0.0075	0.0486	0.0012
Procainamide	156.9188	12.1279	1.1779	0.0861	-0.0098	0.0186	0.0421	0.0024
Quinidine	2.3297	0.1458	1.1988	0.0712	0.0030	0.0142	0.0393	0.0019
Ranolazine	62.1711	11.6969	0.8352	0.0654	-0.1138	0.0585	0.0482	0.0022
Sotalol	4808.5749	1953.7494	4.5806	2.5248	0.8745	0.0117	0.1264	0.0031
Terodiline	2.5254	0.1102	1.8839	0.1122	0.0055	0.0156	0.0591	0.0020
Vandetanib	6.3059	0.3523	1.3719	0.0673	0.0041	0.0147	0.0713	0.0021
Verapamil	0.0825	0.0020	1.5551	0.0523	-0.0059	0.0062	0.0377	0.0010

Additional references

Abi-Gerges, N., T. Indersmitten, K. Truong, W. Nguyen, P. Ratchada, N. Nguyen, G. Page, P. E. Miller, and A. Ghetti. 2020. 'Multiparametric Mechanistic Profiling of Inotropic Drugs in Adult Human Primary Cardiomyocytes', Sci Rep, 10: 7692.

Crumb, W. J., Vicente, J., Johannesen, L., & Strauss, D. G. (2016). An evaluation of 30 clinical drugs against the comprehensive in vitro proarrhythmia assay (CiPA) proposed ion channel panel. Journal of Pharmacological and Toxicological Methods, 81(2), 251–262. https://doi.org/10.1016/j.vascn.2016.03.009

Kramer, J., Obejero-Paz, C. A., Myatt, G., Kuryshev, Y. A., Bruening-Wright, A., Verducci, J. S., & Brown, A. M. (2013). MICE Models: Superior to the HERG Model in Predicting Torsade de Pointes. Scientific Reports, 3(1), 2100. https://doi.org/10.1038/srep02100

Margara, F., Wang, Z. J., Levrero-Florencio, F., Santiago, A., Vázquez, M., Bueno-Orovio, A., & Rodriguez, B. (2021). In-silico human electro-mechanical ventricular modelling and simulation for drug-induced pro-arrhythmia and inotropic risk assessment. Progress in Biophysics and Molecular Biology, 159(xxxx), 58–74. https://doi.org/10.1016/j.pbiomolbio.2020.06.007
